# Enterohemorrhagic *Escherichia coli* O157 outer membrane vesicles administered by oral gavage cause renal tubular injury and acute kidney failure in mice

**DOI:** 10.3389/fcimb.2025.1704731

**Published:** 2025-11-24

**Authors:** Jaromír Háček, Alena Vlková, Daniel Krsek, Lukáš Kolařík, Alžběta Spálenková, Tomáš Sychra, Tereza Tesařová, Emin Gayibov, Radka Václavíková, Jakub Zieg, Martina Bielaszewska

**Affiliations:** 1 Department of Pathology and Molecular Medicine, Second Faculty of Medicine, Charles University, and University Hospital Motol, Prague, Czechia; 2 Department for Welfare of Laboratory Animals, Centre for Toxicology and Health Safety, National Institute of Public Health, Prague, Czechia; 3 National Reference Laboratory for Detection of Infectious Agents by Electron Microscopy, Centre for Epidemiology and Microbiology, National Institute of Public Health, Prague, Czechia; 4 Department of Clinical Hematology, University Hospital Motol, Prague, Czechia; 5 Department of Toxicogenomics, Centre for Toxicology and Health Safety, National Institute of Public Health, Prague, Czechia; 6 Department of General Surgery, Third Faculty of Medicine, Charles University, and University Hospital Královské Vinohrady, Prague, Czechia; 7 Department of Pediatrics, Second Faculty of Medicine, Charles University, and University Hospital Motol, Prague, Czechia; 8 National Reference Laboratory for E. coli and Shigella, Centre for Epidemiology and Microbiology, National Institute of Public Health, Prague, Czechia

**Keywords:** enterohemorrhagic *Escherichia coli* (EHEC), Shiga toxin, outer membrane vesicles, mouse model, oral gavage, hemolytic uremic syndrome, renal tubular damage, acute kidney failure

## Abstract

**Background:**

Outer membrane vesicles (OMVs) secreted by enterohemorrhagic *Escherichia coli* (EHEC) O157 contain Shiga toxin 2 (Stx2), the major virulence factor involved in the pathogenesis of EHEC-associated hemolytic uremic syndrome (EHEC-HUS). However, it remains unclear whether EHEC OMVs produced in the human intestine during infection play a role in EHEC-HUS development. Using a mouse model, we investigated whether EHEC O157 OMVs administered by oral gavage translocate from the gastrointestinal tract to the bloodstream, enter the kidneys, and induce signs of EHEC-HUS. Because mice, unlike humans, express the Stx2 receptor Gb3 on the renal tubular epithelium but not on the glomerular endothelium, we focused on the ability of EHEC O157 OMVs to cause tubular damage, which represents a mechanism that, alongside glomerular thrombotic microangiopathy (TMA), contributes to acute kidney failure in EHEC-HUS.

**Methods:**

The sera and kidneys of BALB/c mice orally administered EHEC O157 OMVs were examined for OMVs by immunoelectron and confocal immunofluorescence microscopy. Histopathological evaluation of the kidneys was performed by light and electron microscopy, and blood analyses were conducted using standard methods. The cytotoxicity of EHEC O157 OMVs toward human renal glomerular endothelial cells (HRGECs) and tubular epithelial cells (HK-2) was determined by Cell Death ELISA. In addition, sera from patients with EHEC O157-associated HUS were examined for O157 OMVs by immunoelectron microscopy.

**Results:**

EHEC O157 OMVs were detected in the sera and kidneys of mice orally administered 100–400 µg of OMVs. The mice exhibited renal tubular epithelial damage and had significantly increased serum creatinine and blood urea nitrogen levels, indicating acute kidney failure. EHEC O157 OMVs induced apoptosis in HRGECs and HK-2 cells, the primary targets in EHEC-HUS. Moreover, EHEC O157 OMVs were found in the sera of patients with EHEC O157-associated HUS.

**Conclusion:**

Orally administered EHEC O157 OMVs translocated from the gastrointestinal tract to the kidneys, where they caused tubular epithelial injury followed by acute kidney failure. Combined with their cytotoxicity toward HRGECs and HK-2 cells and detection in patient sera, these findings indicate that EHEC O157 OMVs contribute to the pathogenesis of EHEC-HUS.

## 1 Introduction

Enterohemorrhagic *Escherichia coli* (EHEC) of serotype O157:H7 is a major cause of EHEC-associated hemolytic uremic syndrome (EHEC-HUS) worldwide (Karch et al., 2005; Tarr et al., 2005; Mellmann et al., 2008; McKee et al., 2020). EHEC-HUS, the triad of nonimmune microangiopathic hemolytic anemia, thrombocytopenia, and acute kidney failure (Tarr et al., 2005), represents the most severe outcome of EHEC infection. It develops as an extraintestinal complication in 10%–15% of children with EHEC diarrhea (Tarr et al., 2005) and is one of the main renal causes of acute kidney failure in childhood (Siegler and Oakes, 2005). EHEC-HUS is a thrombotic microangiopathy (TMA) that primarily affects the renal glomeruli but can also involve the large intestine and the brain (Richardson et al., 1988). In addition to glomerular endothelial injury, renal histopathology in patients with EHEC-HUS also reveals severe tubular epithelial damage (Richardson et al., 1988; Karpman et al., 1998; Porubsky et al., 2014), which substantially contributes to acute kidney failure (Porubsky et al., 2014). The mortality rate of acute EHEC-HUS is 3%–5% (Siegler and Oakes, 2005), and up to 30% of survivors develop late sequelae such as hypertension, proteinuria, neurological complications, and chronic kidney disease (Bláhová et al., 2002; Garg et al., 2003; Rosales et al., 2012).

The major EHEC virulence factors involved in EHEC-HUS pathogenesis are Shiga toxins (Stxs), which are ribosome-inactivating AB_5_ holotoxins (Obrig, 2010; Zoja et al., 2010). During human infection, Stxs are released by EHEC bacteria colonizing the large intestine, absorbed into the circulation, and transported to the kidneys, where they injure microvascular endothelial cells (Obrig, 2010; Zoja et al., 2010) and tubular epithelial cells (Karpman et al., 1998; Porubsky et al., 2014). Stx2, the most common Stx type produced by EHEC strains isolated from HUS patients (Mellmann et al., 2008), is released from the bacteria in two forms: as a free soluble protein and in association with outer membrane vesicles (OMVs) secreted by EHEC (Kolling and Matthews, 1999; Yokoyama et al., 2000; Kunsmann et al., 2015; Bielaszewska et al., 2017). OMVs are nanosized proteoliposomes ubiquitously produced by Gram-negative bacteria that play multiple roles in interbacterial and bacteria–host communication, including the pathogenesis of various diseases (Choi et al., 2015; Park et al., 2017; Caruana and Walper, 2020; Rueter and Bielaszewska, 2020; Díaz-Garrido et al., 2021; Villageliu and Samuelson, 2022; Chen et al., 2023a, 2023b). EHEC OMVs deliver Stx2 and other toxins to human microvascular endothelial and intestinal epithelial cells, causing cellular injury and ultimately apoptosis (Kunsmann et al., 2015; Bielaszewska et al., 2017). In addition, OMV-associated lipopolysaccharide (LPS) and flagellin induce secretion of interleukin-8 from human intestinal epithelial cells (Kunsmann et al., 2015; Bielaszewska et al., 2018), which may further contribute to EHEC-HUS pathogenesis, in which proinflammatory cytokines play key roles (Fitzpatrick et al., 1992; Zoja et al., 2010). Thus, OMVs are potent EHEC virulence tools that may participate in the development of EHEC-HUS.

In order to reach the kidneys during infection, OMVs released by EHEC bacteria in the lumen of the large intestine must cross the intestinal barrier and enter the bloodstream. This translocation has been demonstrated for OMVs derived from *E. coli* laboratory strains (Bittel et al., 2021; Schaack et al., 2024) and intestinal microbiota (Tulkens et al., 2020; Schaack et al., 2022; Chen et al., 2023b), but whether EHEC OMVs can traverse the intestinal barrier *in vivo* has not been investigated. In our previous study, we showed that EHEC O157 OMVs translocated across model intestinal epithelial barriers, including polarized Caco-2 cells and human colonoids (Krsek et al., 2023). In the present study, we used a mouse model to determine whether EHEC O157 OMVs administered by oral gavage can cross the gastrointestinal barrier *in vivo*, reach the kidneys, and elicit signs of HUS. Because mice express the functional Stx receptor Gb3 on renal tubular epithelial cells but not on glomerular endothelial cells (Psotka et al., 2009; Porubsky et al., 2014), we focused on the ability of EHEC O157 OMVs to cause tubular injury—a TMA-independent pathophysiological mechanism that significantly contributes to acute kidney failure in EHEC-HUS (Porubsky et al., 2014). We demonstrate that EHEC O157 OMVs administered to mice by oral gavage translocated across the gastrointestinal barrier into the bloodstream, reached the kidneys, and caused tubular epithelial damage followed by acute kidney failure and, ultimately, death.

## 2 Materials and methods

### 2.1 Isolation and characterization of EHEC O157 OMVs

OMVs were isolated from EHEC O157:H7 strain 5791/99 originating from a patient with HUS (Friedrich et al., 2006). OMVs were collected by ultracentrifugation and purified by OptiPrep (iodixanol; Sigma-Aldrich, Taufkirchen, Germany) density gradient fractionation as described previously (Bielaszewska et al., 2013, 2021). OMV sizes and counts were determined using nanoparticle tracking analysis (NTA) with a NanoSight LM10 instrument (Malvern Panalytical, Great Malvern, UK) as described (Bauwens et al., 2017). OMV protein concentration was quantified with Roti-Nanoquant (Carl Roth, Karlsruhe, Germany). Concentrations of OMV-associated Stx2 and other virulence factors, including cytolethal distending toxin V (CdtV), EHEC hemolysin (EHEC-Hly), and H7 flagellin, were determined using calibration curves generated from purified Stx2, the CdtV-B subunit, EHEC-Hly, and H7 flagellin, respectively, as described previously (Bielaszewska et al., 2013; Kunsmann et al., 2015; Bielaszewska et al., 2017). OMV lipopolysaccharide (LPS) content was measured using the LAL Chromogenic Endotoxin Quantitation Kit (Thermo Fisher Scientific, Prague, Czech Republic) according to the manufacturer’s instructions. The characteristics of purified OMVs from EHEC O157:H7 strain 5791/99 (hereafter referred to as EHEC O157 OMVs) are summarized in **Table 1**.

**Table 1:** Characteristics of OMVs from EHEC O157:H7 strain 5791/99 used in mouse experiments^a^.

OMV diameter NTA (nm)^b^	OMV count (particles/ml x 10^10^)	OMV protein concentration (µg/ml)	O157 LPS (µg/ml)	Stx2 (µg/ml)	CdtV (µg/ml)	EHEC-Hly (µg/ml)	H7 flagellin (µg/ml)
151.7 ± 59.2	2.6 ± 0.9	402 ± 47	916 ± 69	59 ± 18	26 ± 9	1.9 ± 0.5	76 ± 11

a All values are means ± standard deviations from four independent measurements.

b The diameter of EHEC O157:H7 5791/99 OMVs determined by dynamic light scattering ranged from 125.3 nm to 180.8 nm (Bielaszewska et al., 2017).

### 2.2 Mouse experiments

BALB/c mice (specific-pathogen-free, female, age 8–10 weeks, weight 16–22 g) were purchased from Charles River (Sulzfeld, Germany). Mice were housed in cages of four animals each at 24 ± 2 °C with a 12/12-hour light/dark cycle and fed *ad libitum* with a standard diet and water. To facilitate OMV absorption from the gastrointestinal tract, the diet was withdrawn 24 h before the experiments, and the mice received only water. After an acclimatization period of at least 7 days, mice were weighed, and groups of four were administered EHEC O157 OMVs (5 µg, 25 µg, 100 µg, 200 µg, or 400 µg of OMV protein containing 0.73 µg, 3.65 µg, 14.6 µg, 29.2 µg, or 58.4 µg of Stx2, and 11.35 µg, 56.75 µg, 227 µg, 454 µg, or 908 µg of LPS, respectively) by oral gavage using plastic feeding tubes (Instech Laboratories, Plymouth Meeting, PA, USA). Each OMV dose was tested in three independent experiments. Four mice that received PBS instead of OMVs served as negative controls in each experiment. Selection of mice administered OMVs or PBS was done randomly. After administration of OMVs or PBS, mice were monitored daily for clinical symptoms. The severity of clinical symptoms was determined according to a health score modified from the HUS score reported previously (Dennhardt et al., 2018). The health score (0–3) was based on the animals’ activity, reactions, posture, fur condition, and the presence of neurological symptoms (**Supplementary Table S1**). When a mouse’s health status deteriorated, it was euthanized by cervical spine dislocation after inhalation anesthesia with isoflurane. All mice, including PBS-treated controls, were sacrificed 72 h after OMV administration as described above. After weighing, the kidneys, colon, and blood were collected. Blood collection was performed by cardiac puncture after isoflurane anesthesia immediately before cervical spine dislocation.

The animal experiments were performed in a breeding facility accredited by the National Institute of Public Health, Prague, in accordance with the guidelines of the Department for Welfare of Laboratory Animals of the Institute. Throughout the experiments, animals were treated in accordance with the Act of the Czech National Council No. 246/1992 Coll. on the Protection of Animals against Cruelty, as amended.

### 2.3 Histopathological and immunofluorescence examinations

For histopathological examinations, tissue samples (kidney, colon) were fixed in 10% phosphate-buffered formaldehyde (HistoFor; Medesa, Polička, Czech Republic), embedded in paraffin, and sectioned. Sections were deparaffinized and stained with hematoxylin–eosin, periodic acid–Schiff (PAS)/alcian blue, and trichrome using the Ventana BenchMark Special Stains instrument (Roche Diagnostics, Prague, Czech Republic) and examined with a light microscope (Olympus BX53; Olympus Czech Group, Prague, Czech Republic). Tubular damage was semiquantified according to Porubsky et al. (2014). Tubular epithelial cells were first evaluated for the extent of: (a) brush border loss in proximal tubules; (b) epithelial cell flattening; and (c) vacuolization. Each phenomenon was separately scored as follows: 0, absent; 0.5, discretely present; 1, slightly present; 2, moderately present; and 3, severely present. The score for each parameter was calculated as the sum of the percentage representation of each score multiplied by the score itself (resulting in values in the range of 0–300). The pathology score for tubular damage of each mouse was expressed by adding the scores for all three parameters (resulting in values in the range of 0–900).

For immunofluorescence microscopy, cryosections from mouse kidneys prepared after freezing in liquid nitrogen using a cryostat (Leica CM1950; Leica Biosystems, Richmond, IL, USA) were used. Cryosections were stained with a goat anti-mouse fibrinogen antibody conjugated with fluorescein isothiocyanate (Nordic MUbio, Susteren, The Netherlands) and examined with a fluorescence microscope (Olympus BX53).

### 2.4 Transmission electron microscopy of the mouse kidneys

Blocks (1–2 mm³) of kidney tissue were fixed in 4% paraformaldehyde (Thermo Fisher Scientific, Prague, Czech Republic) and postfixed in 2% osmium tetroxide (Sigma-Aldrich, Schnelldorf, Germany). After washing in distilled water, they were dehydrated using a graded (50%–96%) alcohol series, embedded in epoxy resin (Durcupan), and polymerized with epoxy embedding medium (both Sigma-Aldrich, Schnelldorf, Germany) at 56 °C. Ultrathin sections (100 nm) were obtained on a Leica Ultracut EM UC7 ultramicrotome (Leica Biosystems, Richmond, IL, USA), collected on copper formvar/carbon-coated 200-mesh grids (Plano, Wetzlar, Germany), stained with 2% uranyl acetate (Merck, Darmstadt, Germany) and 2% lead citrate (Delta Microscopies, Mauressac, France), and examined with a JEOL JEM-1400 Plus electron microscope (JEOL, Tokyo, Japan). Digital images were acquired using a Megaview G2 Olympus digital camera (Olympus Czech Group, Prague, Czech Republic). The investigator who performed histopathology and transmission electron microscopy of the kidneys was not aware of the mouse allocation to the OMV-treated or PBS-treated groups.

### 2.5 Detection of apoptosis in the mouse kidneys

Approximately 20 mg of kidney tissue from mice administered 5 µg, 25 µg, 100 µg, 200 µg, or 400 µg of EHEC O157 OMVs or PBS (negative control) was homogenized in 200 µL of PBS, and the cell suspension was centrifuged (1600 × g, 20 min) to collect the cell pellet. Apoptotic DNA was isolated from the pellet using the Apoptotic DNA Ladder Extraction Kit (Assay Genie, Dublin, Ireland) according to the manufacturer’s instructions. DNA from a kidney that had been exposed to 1 µM staurosporine (Sigma-Aldrich, Schnelldorf, Germany) for 8 h was used as a positive control. Extracted DNA was loaded onto a 1.2% (v/w) agarose gel (25 µL/lane) and separated by electrophoresis at 5 V/cm for 2.5 h. The gel was stained with Midori Green Advance (Biozym Scientific, Hessisch Oldendorf, Germany) and visualized and photographed using a Gel Stick Imager (INTAS Science Imaging Instruments, Göttingen, Germany).

### 2.6 Hematological and biochemical investigations

Blood for hematological investigations was collected into ethylenediaminetetraacetic acid (EDTA)-treated tubes (MiniCollect Tubes 0.25/0.5 ml, K3EDTA; Dialab, Prague, Czech Republic). Complete blood cell counts (CBC) and white blood cell (WBC) differentials were determined using a Sysmex XN-20 automated hematology analyzer (Sysmex Corporation, Kobe, Japan). The analyzer uses three principles to determine CBCs: the impedance method with hydrodynamic focusing to determine red blood cells (RBCs), platelets, and hematocrit; fluorescence flow cytometry to determine WBCs; and photometric hemoglobin determination based on the cyanmethemoglobin method with a cyanide-free reagent.

Blood for biochemical investigations was collected into Eppendorf tubes without anticoagulant, allowed to clot for 30 min at room temperature, and centrifuged (2000 × g, 15 min, 4°C). Serum was used fresh or frozen at −80°C. Creatinine was determined using the Creatinine Assay Kit, and blood urea nitrogen (BUN) using the Urea Assay Kit (both Sigma-Aldrich, Schnelldorf, Germany) according to the manufacturer’s instructions. Serum concentrations of sodium and potassium were determined using the Sodium Assay Kit (Sigma-Aldrich, Schnelldorf, Germany) and the Potassium Assay Kit (Thermo Fisher Scientific, Prague, Czech Republic), respectively, as recommended by the manufacturers. Lactate dehydrogenase (LDH) was determined using the Lactate Dehydrogenase Activity Assay Kit (Thermo Fisher Scientific, Prague, Czech Republic). All assays were performed in 96-well plates (Sigma-Aldrich, Schnelldorf, Germany), and absorbance was measured with a Multiskan FC microplate reader (Thermo Fisher Scientific, Prague, Czech Republic). The investigators who performed hematological and biochemical investigations were not aware of the mouse allocation to the OMV-treated or PBS-treated groups.

### 2.7 Detection of EHEC O157 OMVs in the mouse sera

Serum samples were centrifuged at 1000 × g for 10 min followed by 12000 x g for 30 min to remove cellular debris (Ou et al., 2023). Subsequently, sera were transferred into polypropylene centrifuge tubes (5 × 20 mm; Beckman Coulter, Inc., Brea, CA, USA) with inserted copper formvar/carbon-coated 400-mesh grids (Ted Pella, Inc., Redding, CA, USA) and centrifuged in an Airfuge ultracentrifuge (Beckman Coulter, Inc., Brea, CA, USA) at 102,000–110,000 × g for 20 min at room temperature. Grids were washed with distilled water, blocked in 1% bovine serum albumin (BSA; Sigma-Aldrich, Schnelldorf, Germany), and stained with rabbit polyclonal anti-*E. coli* O157 lipopolysaccharide (LPS) antibody (Bielaszewska et al., 2017, 2021), followed by goat anti-rabbit IgG conjugated with 10 nm colloidal gold (Sigma-Aldrich, Schnelldorf, Germany). Staining with the secondary antibody alone served as a control for the specificity of OMV immunogold staining (**Supplementary Figure S1A**). After washing with PBS, grids were contrasted with 1% uranyl acetate dihydrate (Merck, Darmstadt, Germany), rinsed with distilled water, and examined using a Hitachi HT7800 electron microscope (Hitachi High-Tech, Tokyo, Japan). Digital images were acquired using a TEM CCD camera AMT XR16 (AMT Imaging, Woburn, MA, USA).

### 2.8 Detection of EHEC O157 OMVs in the mouse kidneys

OMVs in the mouse kidneys were detected by fluorescence confocal laser-scanning microscopy (CLSM) and transmission electron microscopy after immunogold staining. For CLSM, cryosections (prepared as described in section 2.3) were fixed with 4% paraformaldehyde, quenched with 0.2 M glycine (pH 7.2), permeabilized with 0.25% Triton X-100, and blocked with 5% BSA (all Sigma-Aldrich, Schnelldorf, Germany). OMVs were stained with rabbit polyclonal anti-*E. coli* O157 LPS antibody (Bielaszewska et al., 2017, 2021) and Cy3-conjugated goat anti-rabbit IgG (Jackson ImmunoResearch, Cambridge, UK). Staining with Cy3 alone served as a control for the specificity of OMV staining (**Supplementary Figures S1B, C**). Glomerular endothelial cells and tubular epithelial cells were stained with anti-CD31 rat monoclonal antibody (MEC 7.46; Abcam, Cambridge, UK*)* and anti-CD324 (E-cadherin) rat monoclonal antibody (DECMA-1; Thermo Fisher Scientific, Prague, Czech Republic), respectively, followed by goat anti-rat IgG conjugated with Alexa Fluor 488 (Thermo Fisher Scientific, Prague, Czech Republic). Nuclei were stained with 4′,6-diamidino-2-phenylindole (DAPI; Thermo Fisher Scientific, Prague, Czech Republic). Preparations were mounted in a fluorescence mounting medium (Dako, Hamburg, Germany) and analyzed using a confocal laser-scanning microscope Leica TCS SP8 with Acousto-Optical Beam Splitter equipped with Diode 405 nm, Argon, and DPSS 561 nm lasers and an HC PL APO CS2 63x/1.4 immersion oil objective (Leica Microsystems, Wetzlar, Germany). Z-stacks (0.23 µm per slice) were acquired using Leica LAS X version 3.5.7.23225 software (Leica Microsystems, Wetzlar, Germany). 3D images were obtained using the Leica LAS X 3D viewer (Leica Microsystems, Wetzlar, Germany). Images were processed with ImageJ software version 1.53t.

For OMV immunogold staining, copper formvar/carbon-coated 200-mesh grids with renal ultrathin sections (prepared as described in section 2.4) were washed in 0.02 M glycine, blocked with 1% BSA (both Sigma-Aldrich, Schnelldorf, Germany), and incubated with rabbit polyclonal anti-*E. coli* O157 LPS antibody (Bielaszewska et al., 2017, 2021). After washing with 0.1% BSA, the primary antibody was detected with goat anti-rabbit IgG conjugated with 10 nm colloidal gold (Sigma-Aldrich, Schnelldorf, Germany). The grids were washed with PBS, postfixed with 1% glutaraldehyde (Serva, Heidelberg, Germany), contrasted with 2% uranyl acetate dihydrate (Merck, Darmstadt, Germany) and 3% lead citrate (Delta Microscopies, Mauressac, France), rinsed with distilled water, and examined using a Hitachi HT7800 electron microscope (Hitachi High-Tech, Tokyo, Japan). Digital images were acquired using a TEM CCD camera AMT XR16 (AMT Imaging, Woburn, MA, USA).

### 2.9 Interactions of EHEC O157 OMVs with cultured human renal cells

Human renal glomerular endothelial cells (HRGECs; ScienCell Research Laboratories, Carlsbad, CA, USA) were cultured in endothelial cell medium supplemented with 5% fetal bovine serum and 1% endothelial cell growth supplement (all from ScienCell Research Laboratories). Human proximal tubular epithelial cells (HK-2) (Ryan et al., 1994; ATCC, Manassas, VA, USA) were cultured in keratinocyte serum-free medium supplemented with human recombinant epidermal growth factor and bovine pituitary extract (Thermo Fisher Scientific, Prague, Czech Republic). The uptake of EHEC O157 OMVs by the cells was tested as described previously (Bielaszewska et al., 2017). Briefly, cells grown in 96-well plates with black frames (Dialab, Prague, Czech Republic) were incubated for 1–24 h with EHEC O157 OMVs (4 µg/mL of OMV protein) labeled with rhodamine isothiocyanate B-R18 (Thermo Fisher Scientific, Prague, Czech Republic). Fluorescence was measured using a fluorescence plate reader (FLUOstar OPTIMA; BMG Labtech, Ortenberg, Germany) and normalized to the fluorescence of rhodamine isothiocyanate B-R18-labeled OMVs without cells.

Cell death following exposure to EHEC O157 OMVs was determined using the Cell Death Detection ELISA Plus kit (Roche Diagnostics, Mannheim, Germany) as described (Bauwens et al., 2011; Bielaszewska et al., 2017). Briefly, confluent cell cultures grown in 96-well plates (P-LAB, Prague, Czech Republic) were incubated for 72 h with 4 µg/mL of EHEC O157 OMVs (containing approximately 585 ng/mL Stx2), purified Stx2 (Bauwens et al., 2011) (585 ng/mL), 1 µM staurosporine (Sigma-Aldrich, Schnelldorf, Germany), PBS, or remained untreated (negative control). Subsequently, cells were processed using the ELISA kit according to the manufacturer’s instructions to quantify DNA fragments in cell culture supernatants (to determine necrosis) and cell lysates (to determine apoptosis). Optical density at 405 nm (OD_405_) was measured using a Multiskan FC microplate reader (Thermo Fisher Scientific, Prague, Czech Republic), and enrichment factors for apoptosis and necrosis were calculated by dividing the OD_405_ values of sample-treated cells by that of untreated cells

### 2.10 Detection of EHEC O157 OMVs in the sera of patients with EHEC O157-associated HUS

Two patients aged 16 months and 4.5 years were hospitalized at the Department of Pediatrics, University Hospital Motol, Prague, for HUS. EHEC O157:H7 strains harboring the *stx*_2_ gene were detected in the patients’ stool samples using methods described previously (Marejková et al., 2013). Serum samples from the patients were examined for EHEC O157 OMVs by immunoelectron microscopy using rabbit polyclonal anti-*E. coli* O157 LPS antibody and goat anti-rabbit IgG conjugated with 10 nm colloidal gold, as described for mouse sera (section 2.7). Staining with the secondary antibody alone served as a control for the specificity of OMV immunogold staining (**Supplementary Figure S2**). Serum from a child (3.5 years) without EHEC O157 infection was used as a negative control.

### 2.11 Statistical analysis

Data from two or multiple groups were analyzed using the Student’s *t*-test or one-way analysis of variance (ANOVA) with Tukey’s honest significant difference (HSD) or Dunnett’s multiple comparison *post hoc* test, as appropriate. Changes in body weight over time (0 and 72 h) were analyzed by two-way repeated measures (RM) ANOVA followed by Dunnett’s *post hoc* test for comparisons with the control group. Survival was assessed using Kaplan–Meier curves, and statistical significance between groups was evaluated using the log-rank (Mantel–Cox) test. Differences in health scores between groups were analyzed using Fisher’s exact test, and pathology scores were compared using one-way ANOVA with Dunnett’s *post hoc* test versus the control group. *P*-values < 0.05 were considered statistically significant. Statistical analyses were performed using GraphPad Prism software, versions 5.04 and 10.03.

## 3 Results

### 3.1 EHEC O157 OMVs administered by oral gavage translocate from the gastrointestinal tract to the bloodstream and reach the kidneys

**Figure 1:**
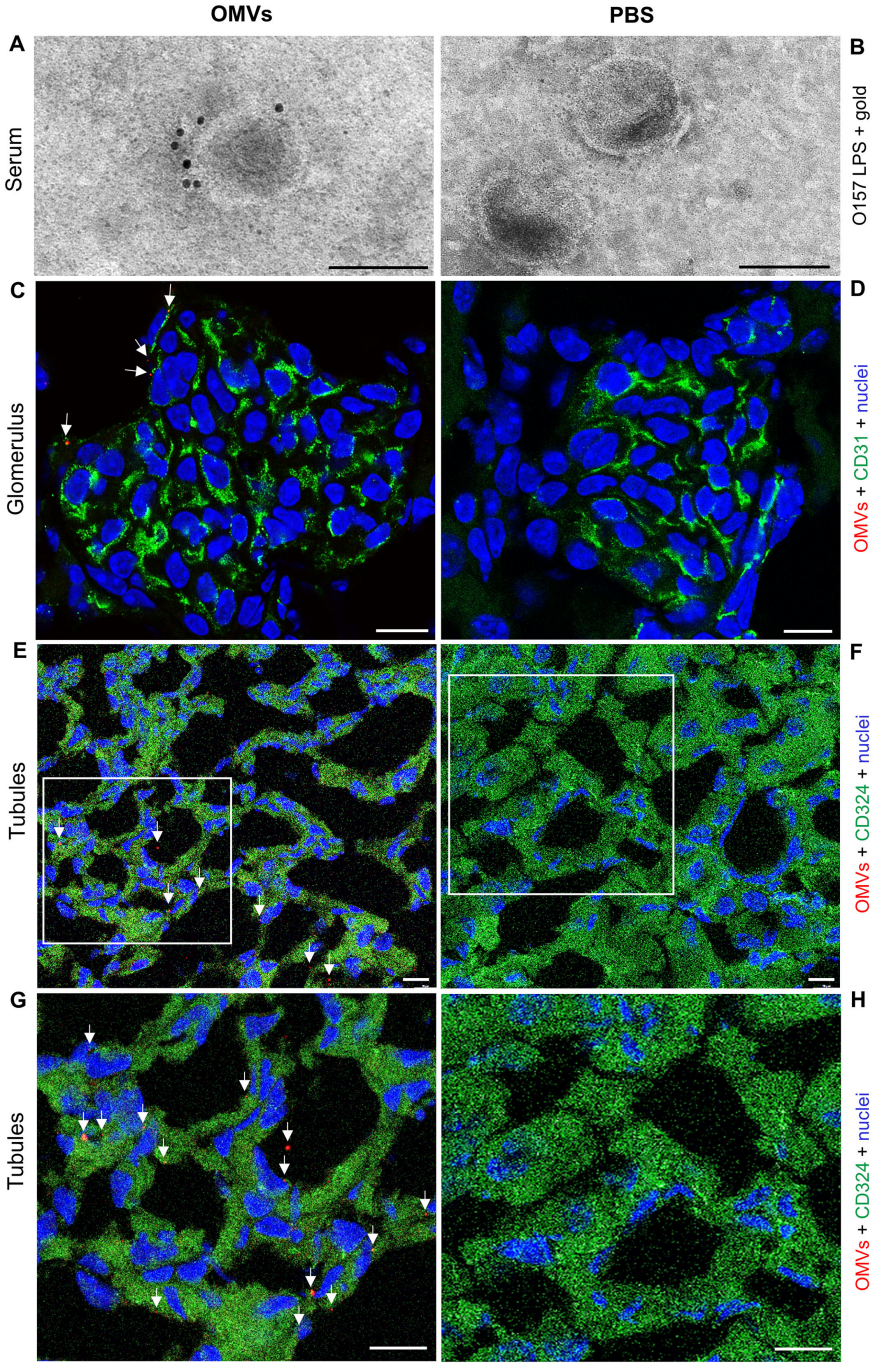
EHEC O157 OMVs administered by oral gavage translocate from the gastrointestinal tract to the bloodstream and reach the kidneys. Detection of EHEC O157 OMVs in the serum **(A, B)**, glomeruli **(C, D)**, and tubules **(E–H)** of mice administered EHEC O157 OMVs **(A, C, E, G)** or PBS **(B, D, F, H)**. **(A, B)** EHEC O157 OMVs in serum samples were detected with rabbit anti-*E. coli* O157 LPS antibody and goat anti-rabbit IgG conjugated with 10 nm colloidal gold. **(C, D)** In the glomeruli, EHEC O157 OMVs (red) were stained with rabbit anti-*E. coli* O157 LPS antibody and Cy3-conjugated goat anti-rabbit IgG, glomerular endothelial cells (green) with anti-CD31 rat antibody and Alexa Fluor 488–conjugated goat anti-rat IgG, and nuclei (blue) with DAPI. **(E, F)** In the tubules, EHEC O157 OMVs (red) were stained with rabbit anti-*E. coli* O157 LPS antibody and Cy3-conjugated goat anti-rabbit IgG, tubular epithelial cells (green) with anti-CD324 rat antibody and Alexa Fluor 488–conjugated goat anti-rat IgG, and nuclei (blue) with DAPI. Panels **(G, H)** show enlarged areas indicated by frames in **(E, F)**, respectively. OMVs in panels **(C)**, **(E)**, and **(G)** are indicated by arrows. Scale bars in panels **(A, B)** are 100 nm; in panels **(C–H)**, 10 µm. Images are representative of findings in mice treated with 100 µg, 200 µg, or 400 µg of EHEC O157 OMVs or with PBS. Crops of representative immunogold-stained and CLSM images are shown. Entire original immunoelectron microscopy images are shown in **Supplementary Figures S3A, B**, and separate red, green, and blue CLSM channels in **Supplementary Figures S4A–D**.

To determine whether EHEC O157 OMVs administered to BALB/c mice by oral gavage translocated from the gastrointestinal tract to the bloodstream and reached the kidneys—a key prerequisite for their involvement in the pathogenesis of EHEC-HUS—we first examined serum samples from OMV-treated mice for the presence of EHEC O157 OMVs using immunoelectron microscopy. We used sera from mice administered 100 µg, 200 µg, or 400 µg of OMVs because these doses caused clinical symptoms and kidney damage, as described below. OMVs stained with anti-*E. coli* O157 LPS antibody and gold-conjugated secondary antibody, confirming their identity as EHEC O157 OMVs, were detected in the sera from OMV-treated mice (**Figure 1A**; **Supplementary Figure S3A**) but not in the sera from PBS-treated control mice (**Figure 1B**; **Supplementary Figure S3B**). Sera from the control mice contained OMV-like structures that did not react with anti-*E. coli* O157 LPS antibody (**Figure 1B**; **Supplementary Figure S3B**) and might have originated from the mouse gut microbiota (Ou et al., 2023).

**Figure 2:**
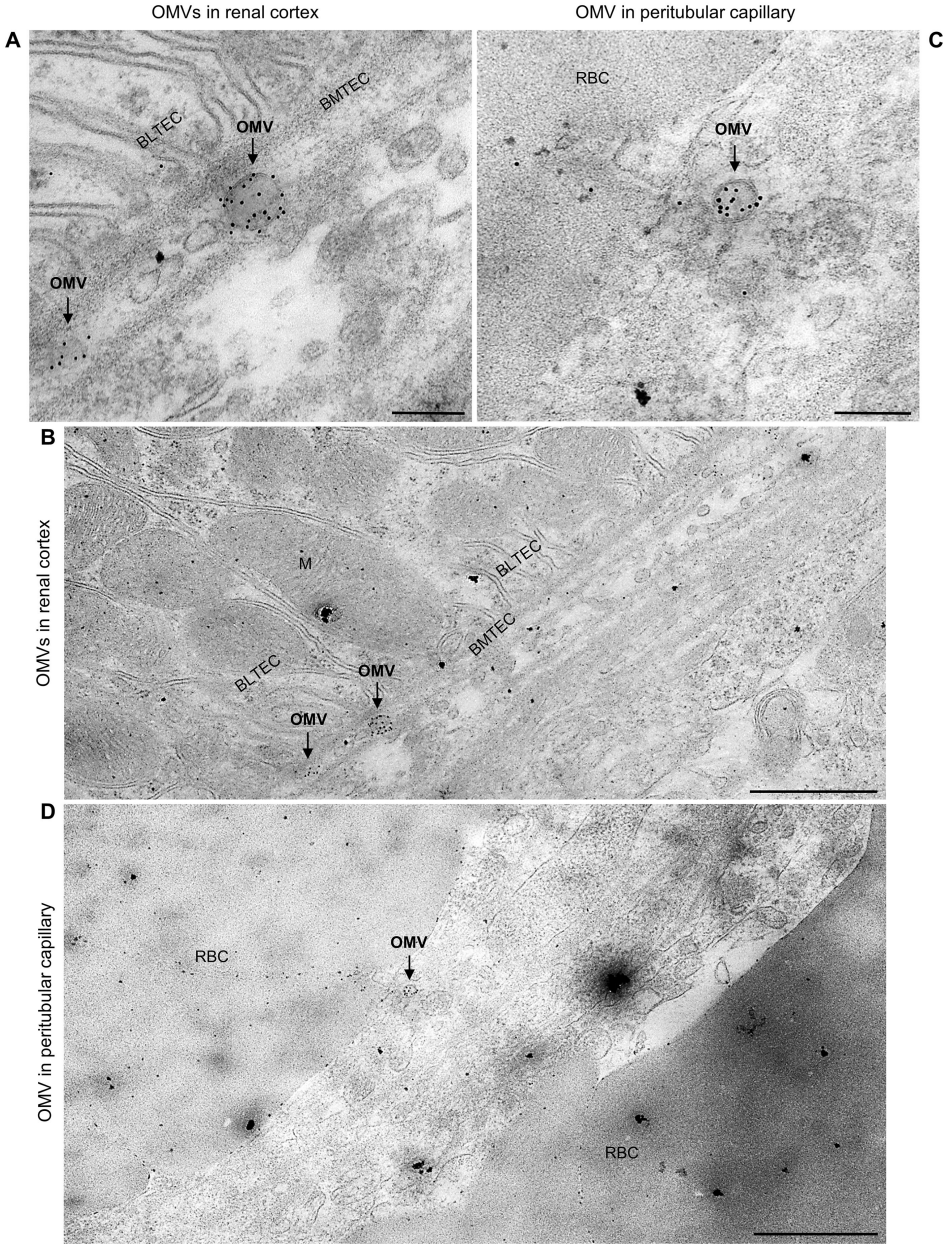
Detection of EHEC O157 OMVs administered by oral gavage in the mouse kidneys by immunoelectron microscopy. Ultrathin sections from the kidneys of two OMV-treated mice stained with rabbit anti-*E. coli* O157 LPS antibody and goat anti-rabbit IgG conjugated with 10 nm colloidal gold. **(A, B)** Mouse treated with 400 µg of OMVs; **(C, D)** mouse treated with 100 µg of OMVs. Panels **(A, C)** (magnification 40,000×; scale bars, 200 nm) show detailed views of immunogold-labeled OMVs (arrows). Panels **(B, D)** (magnification 10,000×; scale bars, 1 µm) show OMVs from panels **(A, C)**, respectively, in context with surrounding structures, demonstrating localization of OMVs (arrows) in the interstitium of the renal cortex near the basement membrane of the tubular epithelial cell (BMTEC) **(B)** and in a peritubular capillary **(D)**. BMTEC, basement membrane of tubular epithelial cell; BLTEC, basal labyrinth of tubular epithelial cell (cell membrane invaginations in the basal part of the cell with numerous mitochondria typical for electrolyte- and water-transporting cells); M, mitochondria; RBC, red blood cell. Crops of representative immunoelectron microscopy images are shown. Entire original images are shown in **Supplementary Figures S6** and **S7**.

To assess whether EHEC O157 OMVs reached the kidneys, we examined kidney sections from the above mice for the presence of OMVs by CLSM and immunoelectron microscopy. CLSM revealed OMVs that reacted with anti-*E. coli* O157 LPS antibody in the glomeruli (**Figure 1C**; **Supplementary Figures S3C**, **S4A**, **S5A**) and in the tubular epithelial cells (**Figures 1E, G**; **Supplementary Figures S3E**, **S4C**, **S5C**). Electron microscopy using immunogold staining with anti-*E. coli* O157 LPS antibody detected EHEC O157 OMVs in the renal cortex near the tubular basement membrane (**Figures 2A, B**; **Supplementary Figures S6A, B**) and in a peritubular capillary (**Figures 2C, D**; **Supplementary Figures S7A, B**). No OMVs reactive with anti-*E. coli* O157 LPS antibody were found in the kidneys of PBS-treated mice by either CLSM (**Figures 1D, F, H**; **Supplementary Figures S3D, F**, **S4B, D**, **S5B, D**) or immunoelectron microscopy (**Supplementary Figure S8**). Altogether, these findings demonstrated that after administration to the gastrointestinal tract, EHEC O157 OMVs crossed the intestinal barrier, entered the bloodstream, and reached the kidneys, thereby enabling their involvement in the pathogenesis of EHEC-HUS.

### 3.2 EHEC O157 OMVs cause signs of disease and death in mice

**Figure 3:**
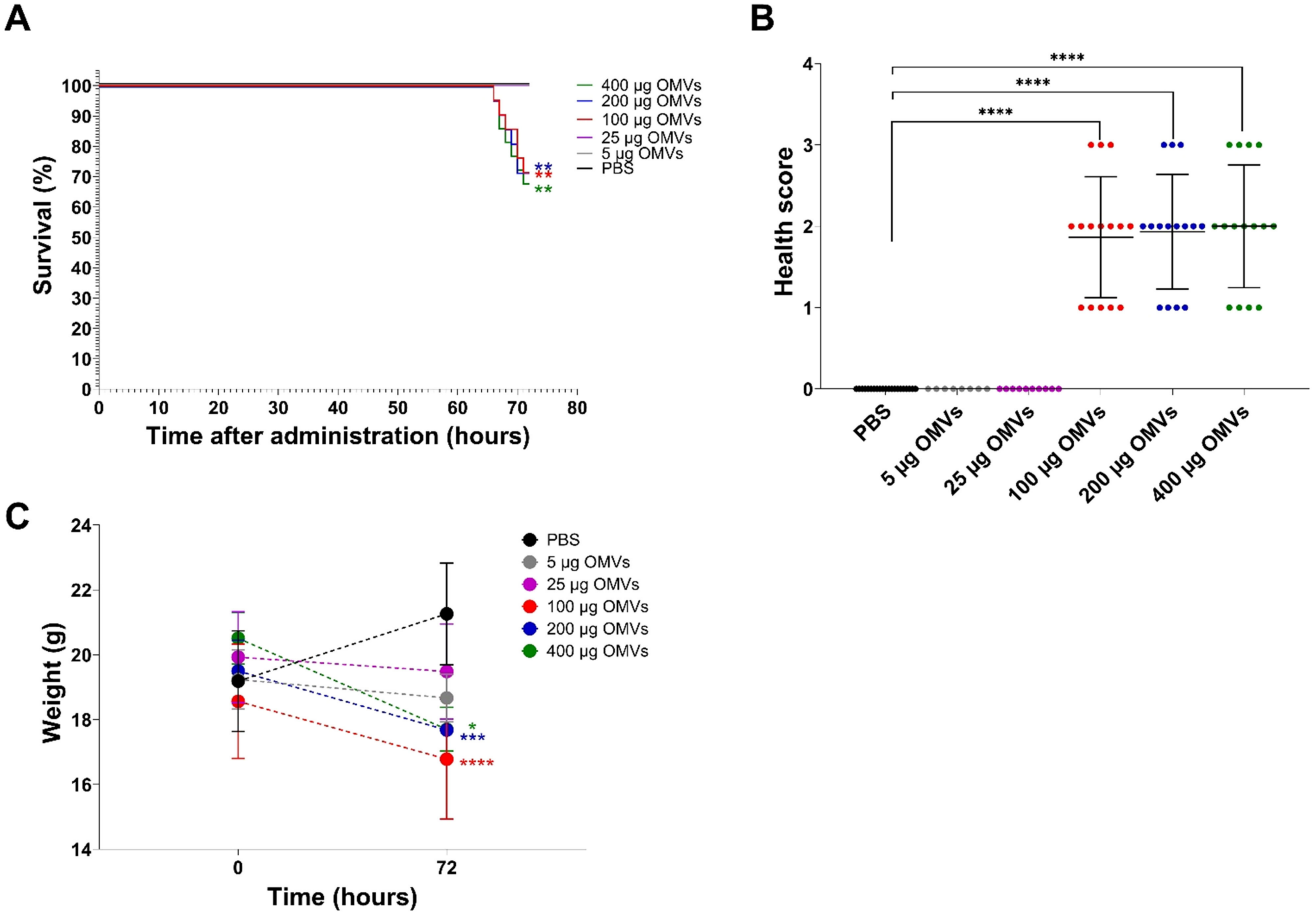
Mouse survival, health status, and weight change 72 h after administration of 5–400 µg of EHEC O157 OMVs or PBS by oral gavage. Data are representative of three experiments. **(A)** Survival curves show the percentage of mice that survived 72 h after administration of 5 µg of OMVs (containing 0.73 µg of Stx2), 25 µg of OMVs (3.65 µg of Stx2), 100 µg of OMVs (14.6 µg of Stx2), 200 µg of OMVs (29.2 µg of Stx2), or 400 µg of OMVs (58.4 µg of Stx2), or PBS. **p < 0.01 for mice treated with 100–400 µg of OMVs compared with mice treated with PBS (log-rank test). **(B)** Health score (0–3) was based on animals’ activity, reactions, posture, fur condition, and presence of neurological symptoms (for details, see **Supplementary Table S1**). Data are means ± standard deviations for groups of mice administered the indicated OMV doses or PBS. ****p < 0.0001 for mice treated with 100–400 µg of OMVs compared with mice treated with PBS (Fisher’s exact test). **(C)** Weight change between time 0 (just before sample administration) and 72 h after administration in mice treated with the indicated OMV doses or PBS. Data are means ± standard deviations for groups of mice administered the indicated OMV doses or PBS. *p < 0.05, ***p < 0.001, and ****p < 0.0001 for mice treated with 400 µg, 200 µg, or 100 µg of OMVs, respectively, compared with mice treated with PBS (two-way RM ANOVA).

To assess whether orally administered EHEC O157 OMVs affect the health of mice, we administered OMVs (5–400 µg of OMV protein) to 82 BALB/c mice by oral gavage and monitored their health status daily. Twenty-one mice that received PBS served as controls. Nineteen of 82 OMV-treated mice, all administered 100–400 µg of OMVs, died between 66 and 71 h after OMV administration (**Table 2**). The survival rates were 71.4% in mice treated with 100 µg or 200 µg of OMVs and 68.2% in mice treated with 400 µg of OMVs (**Figure 3A**; **Table 2**). Forty-five surviving mice treated with 100–400 µg of OMVs developed signs of disease 72 h after administration, including decreased activity, reduced motility, ruffled fur, apathy, lethargy, and sometimes ataxia or tremor, resulting in health scores of 1–3 (**Figure 3B**). The body weight of mice that died or became ill or moribund was significantly decreased after 72 h compared with the weight before OMV administration (time 0) (**Figure 3C**). Eighteen of 82 OMV-treated mice that received 5 µg or 25 µg of OMVs survived until 72 h (**Figure 3A**; **Table 2**). They showed no apparent signs of disease (**Figure 3B**) and experienced only slight, nonsignificant weight loss (**Figure 3C**). All 21 PBS-treated control mice survived (**Figure 3A**; **Table 2**) and remained healthy (**Figure 3B**; **Table 2**) up to 72 h after OMV administration. These data demonstrated that EHEC O157 OMVs administered to BALB/c mice by oral gavage caused disease, the severity of which correlated with the OMV dose.

**Table 2:** Summary of clinical, histopathological, and laboratory findings in BALB/c mice administered EHEC O157 OMVs or PBS by oral gavage.

Sample administered to mice	100 µg OMVs	200 µg OMVs	400 µg OMVs	5 µg OMVs	25 µg OMVs	PBS
OMV-associated Stx2 amount	14.6 µg	29.2 µg	58.4 µg	0.73 µg	3.65 µg	n.a.
No. of mice administered the sample	21	21	22	8	10	21
Number of mice with finding 72 hours after OMV administration						
Death (%)	6 (28.6)	6 (28.6)	7 (31.8)	0 (0)	0 (0)	0 (0)
Survival (%)	15 (71.4)	15 (71.4)	15 (68.2)	8 (100)	10 (100)	21 (100)
Clinical symptoms	15	15	15	0	0	0
Significant weight loss^a^	15	15	15	0	0	0
Tubular damage (histology+TEM)	21	21	22	0	1^b^	0
Glomerular TMA (histology+IF+TEM)	0	0	0	0	0	0
Colitis (histology)	5	5	7	0	0	0
Acute renal failure^c,d^	15	15	15	0	1^b^	0
Thrombocytopenia^d^	0	0	0	0	0	0
Hemolytic anemia^d^	0	0	0	0	0	0
Neutrophilia^d^	15	15	15	8	10	0
Hemoconcentration^d^	15	15	15	8	10	0

a In 45 mice that survived and in 12 mice that died (7 mice that died were not weighed).

b The same mouse; serum creatinine and BUN were 0.67 ± 0.12 mg/dl and 91 ± 12 mg/dl, respectively.

c Significantly increased serum concentrations of creatinine and BUN.

d Hematological and biochemical examinations were performed in mice that survived (blood could not be collected from 19 mice that died).

TEM, transmission electron microscopy; IF, immunofluorescence microscopy; TMA, thrombotic microangiopathy; n.a., not applicable.

### 3.3 EHEC O157 OMVs cause tubular epithelial damage in the mouse kidneys leading to apoptosis

**Figure 4:**
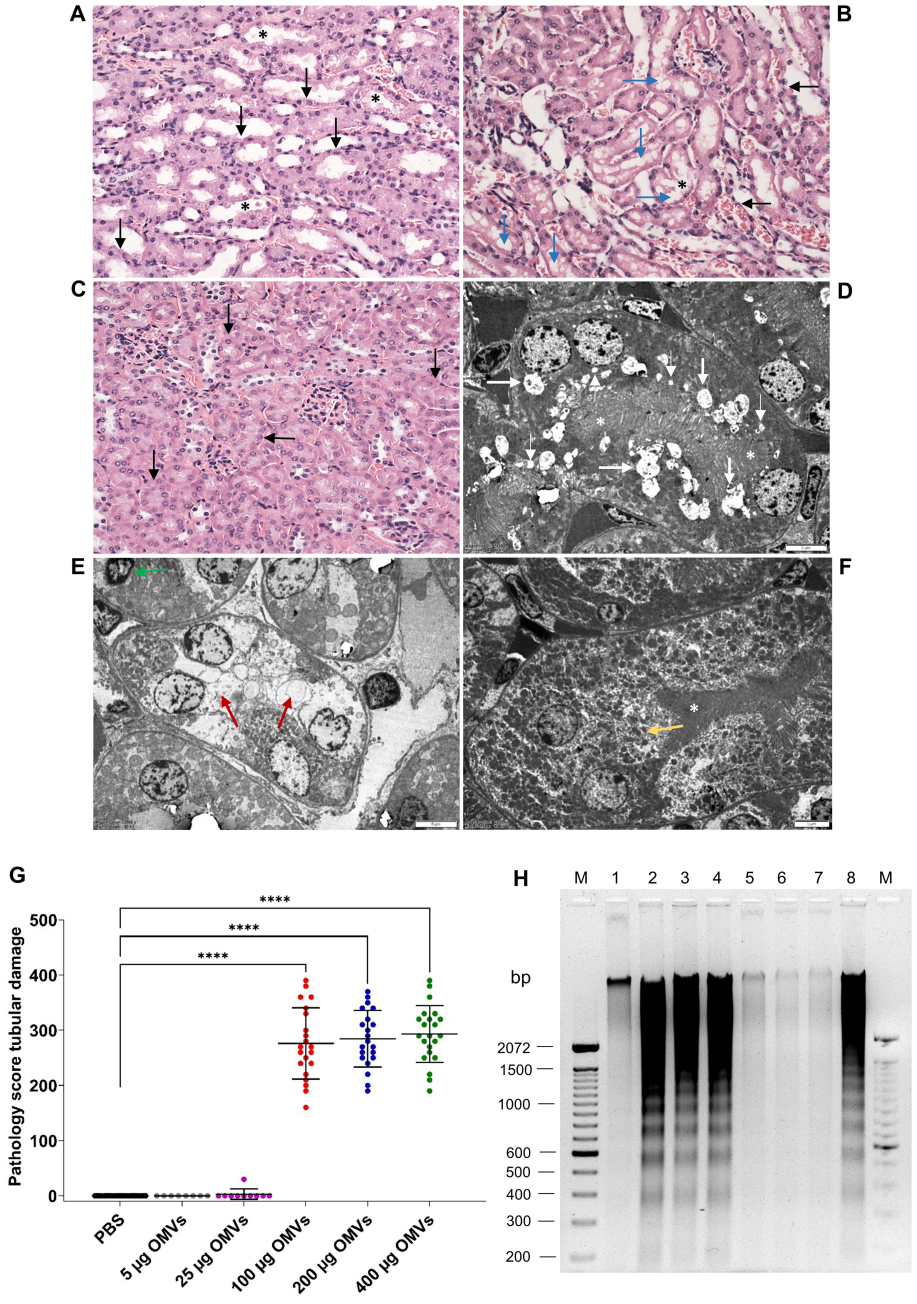
EHEC O157 OMVs administered to mice by oral gavage cause tubular epithelial damage and apoptosis. Histopathology of paraffin sections stained with hematoxylin–eosin **(A–C)** and transmission electron microscopy **(D–F)** of the kidneys from mice treated with 100–400 µg of OMVs **(A, B, D, E)** and from PBS-treated mice **(C, F)**. **(A)** Tubular epithelial cell flattening (black arrows) and intraluminal detached epithelial cells (asterisks). **(B)** Tubular epithelial flattening (black arrows), extensive vacuolization (blue arrows), detached intraluminal epithelial cells (asterisks). **(C)** Normal tubules (arrows) and glomeruli in a PBS-treated mouse. **(D)** Microvacuolization (thin white arrows), macrovacuolization (thick white arrows), and flattening of tubular epithelial cells with a preserved brush border (white asterisks). **(E)** Macrovacuolization of tubular epithelial cells (red arrows) with lost brush border; the green arrow indicates an apoptotic nucleus. **(F)** Normal tubule in a PBS-treated mouse; the yellow arrow indicates a normal tubular epithelial cell and the white asterisk a preserved brush border. **(A–C)** Magnification 400x; **(D–F)** scale bars, 5 µm. Images are representative of findings in mice treated with 100 µg, 200 µg, or 400 µg of EHEC O157 OMVs or PBS. **(G)** Pathology score for tubular epithelial damage was assessed based on the extent of brush border loss, epithelial cell flattening, and vacuolization after 72 h. Data are means ± standard deviations for groups of mice administered the indicated OMV dose or PBS; ****p < 0.0001 for mice treated with 100–400 µg of OMVs compared with mice treated with PBS (one-way ANOVA). **(H)** Apoptosis shown as DNA laddering in the kidneys of mice treated with 100 µg (lane 2), 200 µg (lane 3), or 400 µg (lane 4) of OMVs for 72 h Lanes 1 and 5, kidneys from mice treated with 5 µg and 25 µg of OMVs, respectively. Lanes 6 and 7, kidneys from mice treated with PBS. Lane 8, kidney treated with 1 µM staurosporine (positive control). Lanes M, molecular size marker (100 bp ladder). The entire original gel is shown in **Supplementary Figure S9E**.

We performed histopathological and electron microscopic examinations of the kidneys from OMV-treated mice to determine whether EHEC O157 OMVs caused renal damage. On histopathological examination, all 64 mice that received 100–400 µg of OMVs demonstrated damage to the proximal tubules (**Table 2**), which were dilated and showed regressive epithelial changes, including epithelial cell flattening, vacuolization, and focal desquamation into the tubular lumen (**Figures 4A, B**; **Supplementary Figure S9A**).

In electron microscopy, mice treated with 100–400 µg of OMVs showed extensive microvacuolization and macrovacuolization of the tubular epithelium (**Figures 4D, E**; **Supplementary Figure S9C**), with the brush border being preserved (**Figure 4D**; **Supplementary Figure S9C**) or lost (**Figure 4E**). In some tubules, epithelial cell flattening was observed (**Figure 4D**). Seventeen of 18 mice administered 5 µg or 25 µg of EHEC O157 OMVs did not show any tubular changes on light or electron microscopy (**Table 2**). One mouse that received 25 µg of OMVs showed vacuolization of the tubular epithelium (**Supplementary Figures S10A, B**). None of the 21 control mice that received PBS instead of OMVs had any histopathological or electron microscopic changes in the tubules (**Figures 4C, F**; **Supplementary Figures S9B, D**; **Table 2**). Semiquantification of tubular epithelial damage using the pathology score, based on the extent of brush border loss, epithelial cell flattening, and vacuolization, demonstrated that the damage was significantly higher in mice administered 100–400 µg of OMVs than in control mice administered PBS (**Figure 4G**).

Because tubular epithelial cells in the kidneys of mice treated with 100–400 µg of EHEC O157 OMVs occasionally displayed apoptotic features such as condensed nuclei (**Figure 4E**), we examined the kidneys from OMV-treated mice for DNA fragmentation, a hallmark of apoptosis (Nagata, 2000). The kidneys from mice treated with 100–400 µg of OMVs displayed a typical DNA ladder pattern resulting from internucleosomal DNA cleavage during apoptosis (**Figure 4H**, lanes 2–4; **Supplementary Figure S9E**). A similar DNA pattern was observed in the kidney treated with the apoptosis-inducing agent staurosporine (**Figure 4H**, lane 8). In contrast, no apparent DNA fragmentation was present in kidneys of mice treated with 5 µg or 25 µg of OMVs or with PBS (**Figure 4H**, lanes 1, 5–7). Taken together, these data demonstrated that EHEC O157 OMVs administered to mice by oral gavage caused, in a dose-dependent manner, renal tubular epithelial damage leading to apoptosis.

**Figure 5:**
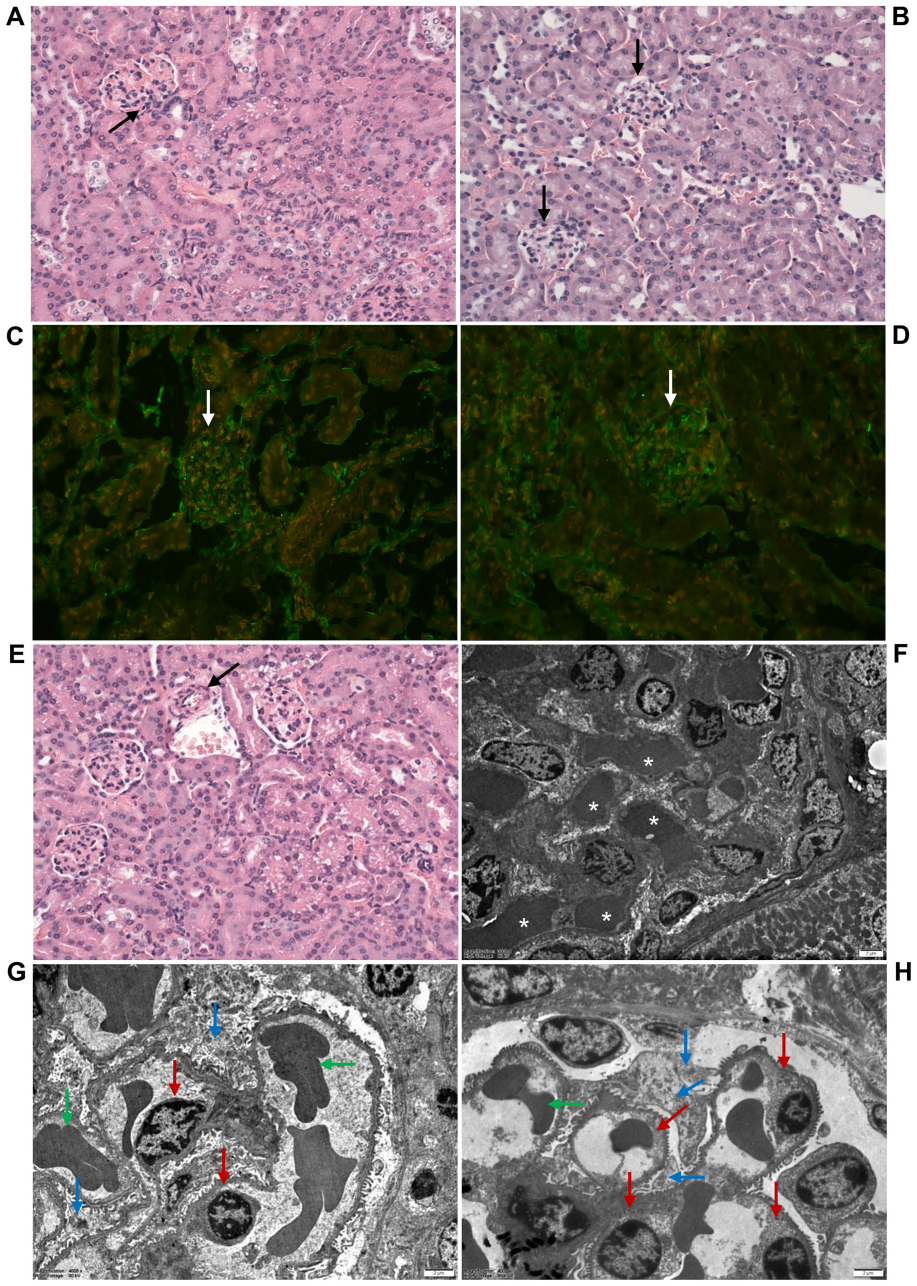
EHEC O157 OMVs administered to mice by oral gavage do not induce glomerular thrombotic microangiopathy. Histopathology of paraffin sections stained with hematoxylin–eosin **(A, B, E)**, immunofluorescence microscopy with anti-fibrinogen antibody **(C, D)**, and transmission electron microscopy **(F–H)** of the kidneys from mice treated with 100–400 µg of OMVs **(A, C, E, F, G)** or with PBS **(B, D, H)**. **(A, F)** OMV-treated mice demonstrated RBC congestion in the glomerular capillaries [arrow in **(A)**; white asterisks in **(F)**], but no capillary fibrin microthrombi were detected **(C, G)**. **(C)** Glomerulus without microthrombi (arrow). **(G)** Normal glomerular endothelial cells (red arrows), podocytes (blue arrows), and single RBCs in capillary lumen (green arrows). **(E)** Arteriole (arrow) with a free lumen without thrombus. No histopathological and electron microscopic abnormalities were found in the glomeruli of control mice **(B, D, H)**. **(B, D)** Normal glomeruli (black and white arrows, respectively). **(H)** Normal glomerular endothelial cells (red arrows), podocytes (blue arrows), and single RBCs in capillary lumen (green arrows). **(A–E)** Magnification 400×; **(F–H)** scale bars, 2 µm. Images are representative of findings in mice treated with 100 µg, 200 µg, or 400 µg of EHEC O157 OMVs or PBS.

The glomeruli of mice administered 100–400 µg of OMVs showed, on histopathological examination, RBC capillary congestion (**Figure 5A**), but no fibrin microthrombi were detected in the capillaries by histopathology or by immunofluorescence staining with anti-fibrinogen antibody (**Figure 5C**). There were no pathological changes in the renal arterioles and arteries (**Figure 5E**). The renal interstitium was congested, with no inflammatory infiltration or fibrosis (**Figure 5A**). By electron microscopy, RBC congestion in the glomerular capillaries was sometimes observed (**Figure 5F**), but no microthrombi were detected (**Figure 5G**). There were no pathological changes in the glomerular endothelium or podocytes (**Figure 5G**). None of the 18 mice administered 5 µg or 25 µg of EHEC O157 OMVs, and none of the 21 PBS-treated control mice, showed any histopathological or electron microscopic changes in the renal glomeruli (**Figures 5B, D, H**; **Table 2**).

### 3.4 EHEC O157 OMVs cause acute kidney failure in mice

**Figure 6:**
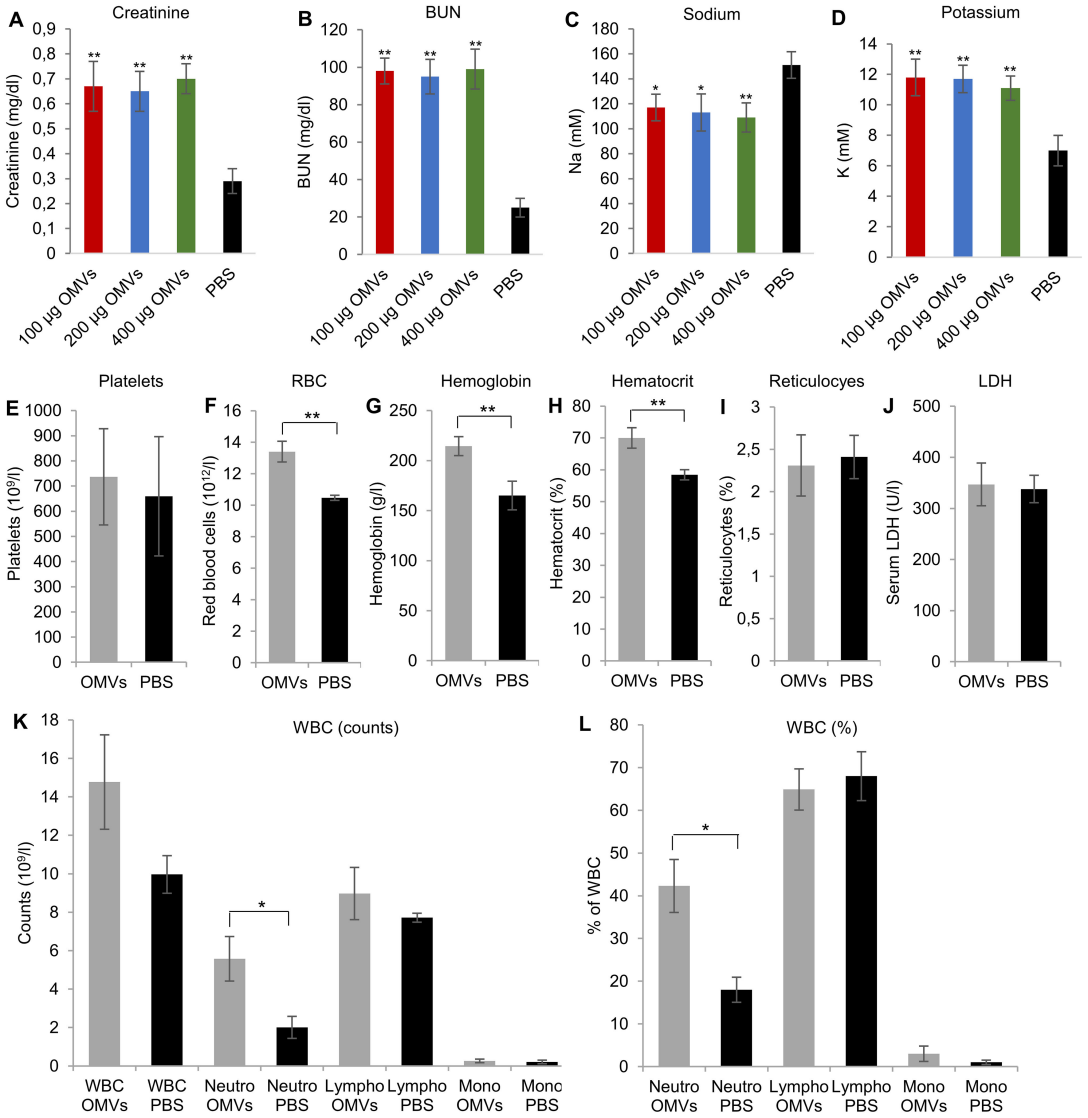
EHEC O157 OMVs administered by oral gavage cause acute renal failure but not thrombocytopenia and hemolytic anemia in mice. Serum concentrations of **(A)** creatinine, **(B)** blood urea nitrogen (BUN), **(C)** sodium, and **(D)** potassium in mice treated with the indicated doses of OMVs or PBS. **(E–L)** Hematological findings in mice treated with OMVs or PBS, including **(E)** platelet counts, **(F)** red blood cell counts, **(G)** hemoglobin concentration, **(H)** hematocrit, **(I)** percentage of reticulocytes, **(J)** serum LDH concentration, **(K)** total white blood cell (WBC) counts and total counts of neutrophils, lymphocytes, and monocytes, and **(L)** WBC differential. Neutro, neutrophils; lympho, lymphocytes; mono, monocytes. Data are means ± standard deviations from the values in OMV-treated mice (OMVs) and PBS-treated mice (PBS). **p < 0.01 or *p < 0.05 for differences between OMV-treated and PBS-treated mice (one-way ANOVA with Tukey’s HSD and Student’s *t*-test).

To evaluate the impact of the tubular epithelial damage induced by EHEC O157 OMVs on kidney function, we measured concentrations of creatinine and BUN in the sera from mice that did or did not develop tubular damage after OMV treatment. Mice treated with 100–400 µg of OMVs that developed tubular damage (**Figures 4A, B, D, E**; **Supplementary Figures S9A, C**) and from whom blood was available for examination had significantly increased serum creatinine and BUN concentrations compared with control mice (**Figures 6A, B**). Moreover, they had significantly decreased sodium and significantly increased potassium concentrations in the sera (**Figures 6C, D**), indicating severe tubular dysfunction.

In contrast, among 18 mice treated with 5 µg or 25 µg of OMVs, only one mouse that received 25 µg of OMVs and showed vacuolization of the tubular epithelium (**Supplementary Figures S10A, B**) had significantly increased creatinine (0.67 ± 0.12 mg/dl) and BUN (91 ± 12 mg/dl) (**Table 2**). In the remaining 17 mice, in which no tubular epithelial damage was detected microscopically, serum concentrations of creatinine and BUN did not differ from those in PBS-treated mice (**Supplementary Figures S11A, B**).

These findings demonstrated that EHEC O157 OMVs in doses capable of damaging tubular epithelium caused acute kidney failure in mice and that OMV-mediated tubular epithelial damage alone, without glomerular TMA, was sufficient to induce kidney failure. Moreover, the correlation between the OMV ability to cause tubular epithelial damage, acute kidney failure, and disease symptoms including death (**Table 2**) suggests that OMV-mediated acute kidney failure was responsible for the clinical symptoms observed in OMV-treated mice.

### 3.5 Colitis is not necessary for EHEC O157 OMV-mediated tubular damage

Because colitis frequently occurs in patients with EHEC O157 infection (Griffin et al., 1990; Kelly et al., 1990), we investigated whether OMV-treated mice developed colitis. Histopathological examination of the colon demonstrated that 17 of 64 mice that received 100–400 µg of OMVs (26.6%) developed colitis (**Table 2**), characterized by inflammatory infiltration of the mucosa, mucosal erosions, and ulcerations (**Supplementary Figures S12B, C**). Colitis was not observed in any of the 18 mice that received 5 µg or 25 µg of OMVs (**Table 2**). The colons from PBS-treated mice did not show any histopathological changes (**Supplementary Figure S12A**).

These findings indicated that although colitis development in mice increases intestinal permeability and enables OMV translocation from the intestine to the circulation (Ou et al., 2023), this pathology was not necessary for OMVs to induce renal tubular damage in mice.

### 3.6 EHEC O157 OMVs cause hemoconcentration and neutrophilia but not thrombocytopenia and hemolytic anemia in mice

Consistent with the absence of glomerular TMA (**Figures 5C, G**), there was no decrease in platelet counts in EHEC O157 OMV-treated mice compared with control mice (**Figure 6E**). Moreover, OMV-treated mice showed no decrease in RBC counts or hemoglobin concentration and no increase in reticulocyte counts or serum LDH (**Figures 6F, G, I, J**), demonstrating the absence of hemolytic anemia. In fact, RBC counts, hemoglobin concentration, and hematocrit were significantly increased in OMV-treated mice compared with control mice (**Figures 6F, G, H**), suggesting dehydration and hemoconcentration.

Regarding WBCs, OMV-treated mice demonstrated significantly increased total counts and percentages of neutrophils compared with control mice, while lymphocytes and monocytes did not significantly differ between the two groups (**Figures 6K, L**). Taken together, due to the inability of EHEC O157 OMVs to induce TMA in Gb3-negative mouse glomeruli, the OMVs did not cause thrombocytopenia or hemolytic anemia in mice. Instead, they caused neutrophilia and hemoconcentration.

### 3.7 EHEC O157 OMVs cause apoptosis of human renal glomerular endothelial cells and tubular epithelial cells *in vitro*

**Figure 7:**
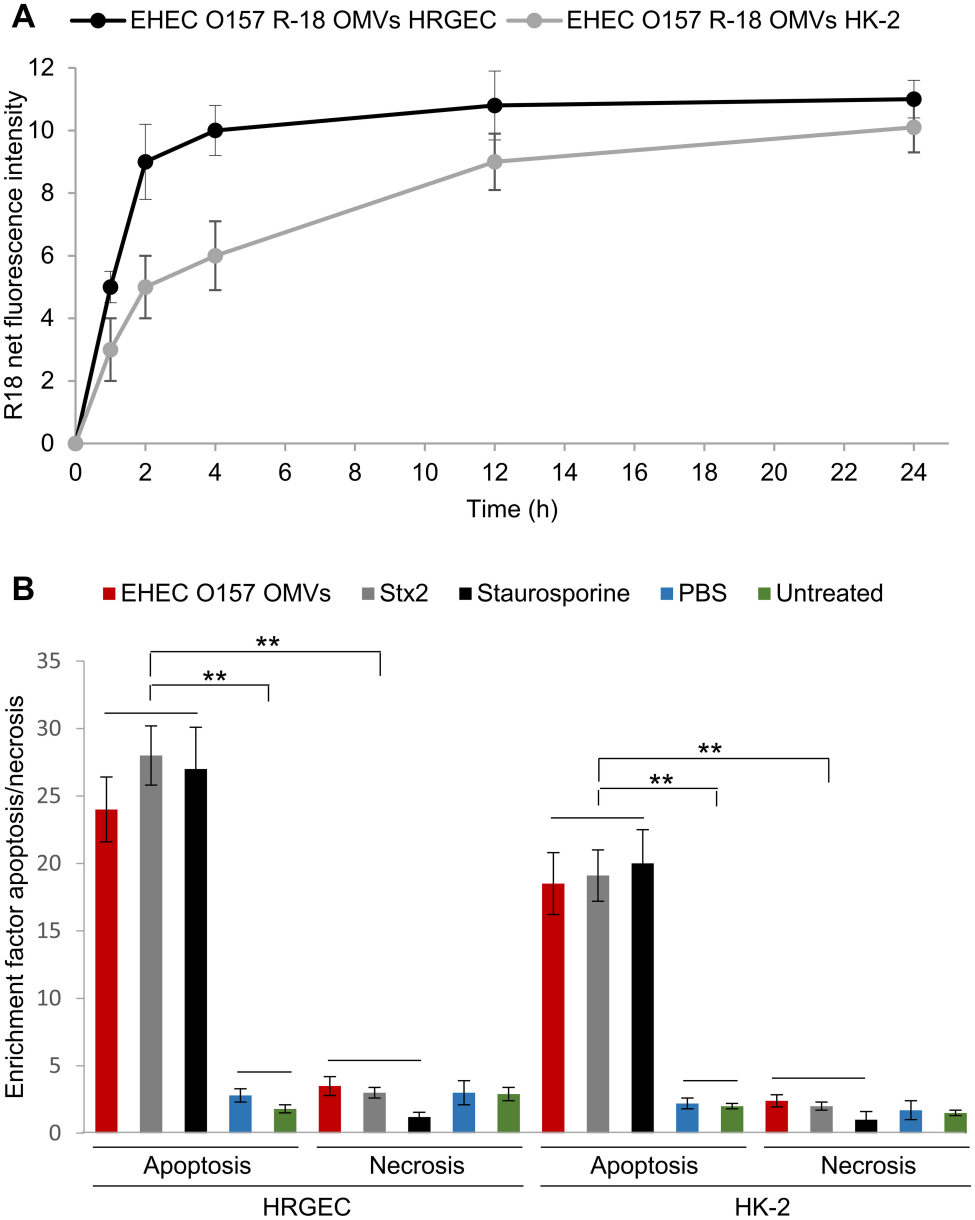
EHEC O157 OMVs are taken up by and cause apoptosis of human renal glomerular endothelial cells (HRGEC) and proximal tubular epithelial cells (HK-2). **(A)** Time-dependent uptake of EHEC O157 OMVs labeled with rhodamine isothiocyanate B-R18 (EHEC O157 R18-OMVs) by HRGEC and HK-2 cells. R-18 net fluorescence is the fluorescence of cells incubated with EHEC O157 R18-OMVs normalized to the fluorescence of EHEC O157 R-18 OMVs without cells. **(B)** Apoptosis and necrosis caused by EHEC O157 OMVs in HRGEC and HK-2 cells after 72 h of incubation as determined by Cell Death Detection ELISA. Enrichment factors were calculated by dividing OD_405_ values of sample-treated cells by those of untreated cells. Data are means ± standard deviations from three experiments. **p < 0.01 for differences between apoptosis of cells exposed to EHEC O157 OMVs, Stx2, or staurosporine on one hand, and to PBS or no treatment on the other; and between apoptosis and necrosis caused by EHEC O157 OMVs, Stx2, and staurosporine (one-way ANOVA with Tukey’s HSD).

We further investigated whether EHEC O157 OMVs interact with human renal glomerular endothelial cells and tubular epithelial cells, which both contain Gb3 (Porubsky et al., 2014; Legros et al., 2017) and are major targets of Stx2 during EHEC-HUS. We found that EHEC O157 OMVs labeled with rhodamine isothiocyanate B-R18 (EHEC O157 R18-OMVs) were taken up by primary human renal glomerular endothelial cells (HRGECs) and the proximal tubular epithelial cell line HK-2 in a time-dependent manner (**Figure 7A**). After 72 h of incubation with the cells, EHEC O157 OMVs induced significant apoptosis in both HRGEC and HK-2 cells, the extent of which was similar to that caused by purified Stx2 (585 ng/mL, present in OMVs) and staurosporine used as a positive control (**Figure 7B**). In contrast, necrosis induced by EHEC O157 OMVs was comparable to that caused by PBS and to the background necrosis of untreated cells (**Figure 7B**).

Altogether, these findings demonstrated that EHEC O157 OMVs interacted with HRGEC and HK-2 cells and caused their death via apoptosis, which was largely mediated by Stx2. The OMVs may thus be involved in the injury of human renal glomerular endothelial and tubular epithelial cells in patients with EHEC-HUS.

### 3.8 EHEC O157 OMVs are present in the sera of patients with EHEC O157-associated HUS

**Figure 8:**
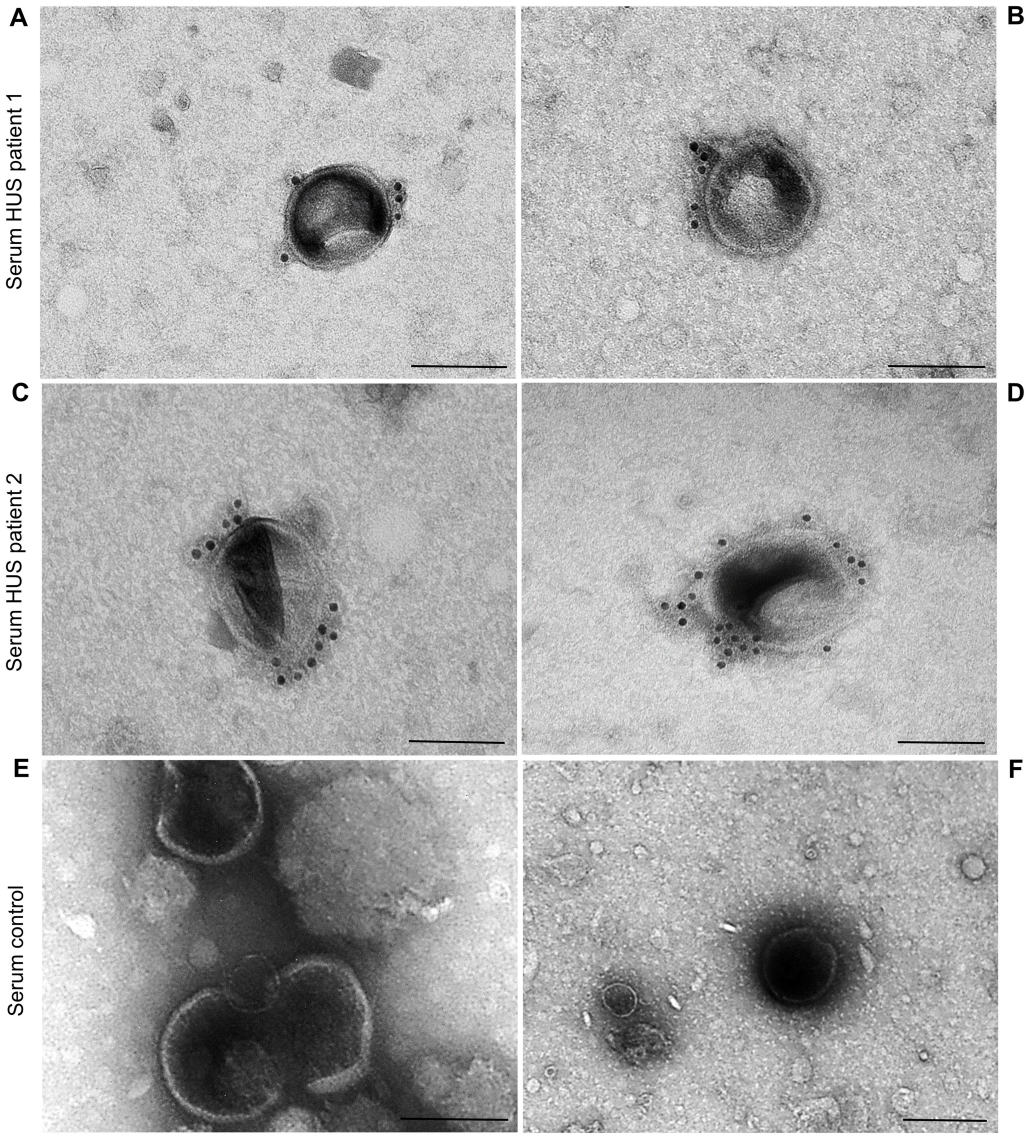
EHEC O157 OMVs are present in the sera from patients with HUS caused by EHEC O157:H7 strains, but not of a person without EHEC O157 infection. Immunoelectron microscopy of serum samples from HUS patient 1 **(A, B)**, HUS patient 2 **(C, D)**, and a control person without EHEC O157 infection **(E, F)**. EHEC O157 OMVs were detected with rabbit anti-*E. coli* O157 LPS antibody and goat anti-rabbit IgG conjugated with 10 nm colloidal gold. Scale bars, 100 nm. Crops of representative immunoelectron microscopy images are shown. Entire original images are shown in **Supplementary Figure S13**.

To determine whether EHEC O157 OMVs produced in the human intestine during EHEC infection (Bauwens et al., 2017) translocate to the bloodstream, we examined serum samples from two pediatric patients with HUS caused by EHEC O157:H7 strains for the presence of EHEC O157 OMVs. OMVs that reacted with anti-*E. coli* O157 LPS antibody were detected in the sera of both patients (**Figures 8A–D**; **Supplementary Figures S13A–D**) but not in the serum from a control child without EHEC O157 infection (**Figures 8E, F**; **Supplementary Figures S13E, F**). Consistent with the findings in mice, this strongly supports the involvement of EHEC O157 OMVs in the pathogenesis of EHEC-HUS in humans.

## 4 Discussion

In recent decades, membrane vesicles secreted by various bacterial species have been implicated in the pathogenesis of a number of infectious and non-infectious diseases (Rueter and Bielaszewska, 2020; Wei et al., 2020; Zhang et al., 2021; Han et al., 2022; Chen et al., 2023a, 2023b; Xie et al., 2023a, 2023b; Wang et al., 2023; Liang et al., 2024; Liu et al., 2024; Olovo et al., 2024; Liu et al., 2025; Wei et al., 2025). OMVs from EHEC O157:H7 carry approximately 50% of Stx2, the major virulence factor produced by EHEC bacteria, while the other 50% of the toxin is released as a free, OMV-unbound protein (Bielaszewska et al., 2017). Although the role of free Stx2 in the pathogenesis of EHEC-HUS has been demonstrated in numerous studies that applied to experimental animals purified Stx2 alone or with LPS (Tesh et al., 1993; Palermo et al., 2000; Keepers et al., 2006; Rasooly et al., 2010; Porubsky et al., 2014; Dennhardt et al., 2018), the pathogenetic involvement of Stx2-containing OMVs is poorly understood. In the only study that addressed this issue (Kim et al., 2011), OMVs were administered to mice intraperitoneally, allowing them to bypass the intestinal barrier, which they must overcome during EHEC infection. To the best of our knowledge, our study is the first that investigated the involvement of EHEC OMVs in the pathogenesis of EHEC-HUS by administering OMVs to the gastrointestinal tract, where they are produced during EHEC infection.

Using the mouse model, with its limitations, we demonstrated that EHEC O157 OMVs meet the requirements for participation in the pathogenesis of EHEC-HUS. Specifically, after administration to the mouse gastrointestinal tract, EHEC O157 OMVs translocated to the bloodstream (**Figure 1A**; **Figures 2C, D**) and reached the kidneys, where they localized in glomerular endothelial cells (**Figure 1C**; **Supplementary Figure S3C**) and tubular epithelial cells (**Figures 1E, G**; **Supplementary Figure S3E**). Due to the absence of the Stx2 receptor Gb3 in mouse glomerular endothelial cells (Psotka et al., 2009; Porubsky et al., 2014), OMV-treated mice did not develop Stx2-mediated glomerular endothelial damage and the associated glomerular TMA (**Figures 5C, G**), thrombocytopenia, and hemolytic anemia (**Figures 6E–J**), which are hallmarks of EHEC-HUS in humans (Tarr et al., 2005), who possess Gb3 on the glomerular endothelium (Obrig, 2010; Porubsky et al., 2014; Legros et al., 2017). However, mice orally treated with EHEC O157 OMVs developed, like patients with EHEC-HUS (Karpman et al., 1998; Porubsky et al., 2014), severe tubular epithelium injury (**Figures 4A, B, D, E**), which led to apoptosis (**Figure 4H**) and was followed by acute kidney failure (**Figures 6A, B**), a defining characteristic of EHEC-HUS (Tarr et al., 2005). Our observation that tubular epithelial injury and kidney failure were induced by 100–400 µg of OMVs containing 14.6–58.4 µg of Stx2 (**Table 2**), but not by lower OMV doses (containing 0.73–3.65 µg of Stx2), is in agreement with a study in which 50 µg of purified Stx2 administered by oral gavage was required to induce tubular apoptosis and death in mice, while lower toxin doses (0.5 µg and 1 µg) failed to do so (Rasooly et al., 2010).

It should be emphasized that EHEC O157 OMVs are complex structures that carry, in addition to Stx2, several other putative EHEC virulence proteins and LPS (**Table 1**). EHEC O157 OMV-associated LPS and flagellin induce secretion of interleukin-8 from human intestinal epithelial cells (Bielaszewska et al., 2018), which may also contribute to the pathogenesis of EHEC-HUS (Zoja et al., 2010). Although our observation that Gb3-positive renal tubular epithelial cells were major targets of OMV-mediated injury in mice is consistent with a specific Stx2-mediated effect, experiments with OMVs from a *stx*_2_-deletion mutant should be performed in a future study. This would allow to determine the specific contribution of OMV-associated Stx2 to the disease phenotype observed in EHEC O157 OMV-treated mice and distinguish it from the contributions of other OMV-associated virulence factors, in particular LPS, which potentiates pathological effects of Stx2 in a mouse model (Keepers et al., 2006).

Our observation that EHEC O157 OMVs caused acute kidney failure in mice through tubular epithelial damage, without inducing glomerular TMA—which underlies acute kidney failure in EHEC-HUS patients (Tarr et al., 2005; Obrig, 2010)—is in agreement with studies that used purified Stx2 in mice (Palermo et al., 2000; Porubsky et al., 2014) or oral infection with Stx2-producing EHEC strains (Wadolkowski et al., 1990; Mohawk et al., 2010). Porubsky and colleagues demonstrated, using transgenic mice lacking Gb3 in tubular epithelial cells, that the tubular absence of Gb3 protected mice against Stx2-mediated tubular injury, acute kidney failure, and death observed in wild-type mice that carried Gb3 on tubular epithelial cells (Porubsky et al., 2014). Neither the wild-type nor the tubular Gb3-lacking mice developed glomerular endothelial injury and TMA (Porubsky et al., 2014). Based on these observations, the authors concluded that tubular epithelial damage represents a separate pathophysiological mechanism that importantly contributes to Stx2-mediated acute kidney failure, most likely due to electrolyte disturbance (Porubsky et al., 2014), which also occurred in our mice treated with EHEC O157 OMVs (**Figures 6C, D**). A clinical parallel to this observation in mice was the authors’ finding that two of 10 patients with EHEC-HUS who developed acute kidney failure did not have glomerular TMA, but all of them displayed renal tubular injury (Porubsky et al., 2014).

In addition to the ability of EHEC O157 OMVs to reach and injure kidneys after oral administration to mice, two additional findings in our study support their causative role in the pathogenesis of EHEC-HUS in humans. First, the OMVs induced apoptosis of human glomerular endothelial cells and tubular epithelial cells *in vitro* (**Figure 7B**), demonstrating their toxic effect toward the major target cells affected during EHEC-HUS. A similar extent of apoptosis induced by Stx2-containing EHEC O157 OMVs and by purified Stx2 in the amount present in OMVs (**Figure 7B**) indicated that Stx2 was the major OMV component responsible for the apoptosis of glomerular endothelial cells and tubular epithelial cells. This is in accordance with our previous observation in human microvascular endothelial cells (Bielaszewska et al., 2017) and with the report that purified Stx2 induced a dose- and time-dependent apoptosis of HK-2 cells (Porubsky et al., 2014). The toxicity of OMV-associated Stx2 toward human glomerular endothelial and tubular epithelial cells leading to their apoptosis is consistent with the presence of Gb3 in both cell types (Porubsky et al., 2014; Legros et al., 2017). Second, we detected EHEC O157 OMVs in the serum samples of pediatric patients with HUS caused by EHEC O157:H7 strains (**Figures 8A–D**). This demonstrates that, as in mice, in humans EHEC O157 OMVs translocate from the intestine, where they are produced during EHEC infection (Bauwens et al., 2017), to the bloodstream, which enables them to reach the kidneys. It should be emphasized that staining of the serum OMVs in these HUS patients (as well as in OMV-treated mice) with an antibody against *E. coli* O157 LPS, which is the major component of EHEC O157 OMVs (**Table 1**), confirms that the OMVs were derived from EHEC O157 bacteria. This specific staining enables differentiation of EHEC O157 OMVs in the sera from HUS patients and OMV-treated mice from other vesicular structures such as exosomes and microvesicles originating from host cells (Ståhl et al., 2019; Karpman and Tontanahal, 2021) and from OMVs derived from other bacteria, e.g., intestinal microbiota (Jones et al., 2020; Schaack et al., 2022). Our findings of EHEC O157 OMVs in the sera of patients with EHEC O157-associated HUS and in the sera of mice orally administered EHEC O157 OMVs extend previous reports on the detection of OMVs from nonpathogenic bacteria, mostly intestinal microbiota, in the blood of humans with disrupted (Tulkens et al., 2020) or intact intestinal barriers (Schaack et al., 2022) and of mice orally administered OMVs (Jones et al., 2020) or colonized with OMV-producing bacteria (Bittel et al., 2021; Ou et al., 2023).

Although, due to the absence of Gb3 in the mouse glomerular endothelium, EHEC O157 OMVs did not cause thrombocytopenia or hemolytic anemia in mice (**Figures 6E–J**), the OMV-treated mice developed hemoconcentration and neutrophilia (**Figures 6F–H, K, L**), which are poor prognostic markers in patients with EHEC-HUS (Walters et al., 1989; Fitzpatrick et al., 1992; Ardissino et al., 2015; Loos et al., 2021). Hemoconcentration and neutrophilia were also observed in mice treated with purified Stx2 (Dennhardt et al., 2018). Hemoconcentration at EHEC-HUS onset is a predictor of severe ischemic organ damage and central nervous system involvement with neurological complications (Ardissino et al., 2015; Alconcher et al., 2018; Loos et al., 2021), which are the most common cause of death in the acute phase of EHEC-HUS (Gerber et al., 2002; Zieg et al., 2012; Alconcher et al., 2018). Moreover, hemoconcentration was a risk factor for severe long-term sequelae following the acute phase (Ardissino et al., 2015). Early volume expansion in patients with EHEC-HUS was shown to be essential for reducing ischemic organ damage and improving short-term and long-term outcomes (Ake et al., 2005; Ardissino et al., 2016; Böckenhauer et al., 2024). In addition to hemoconcentration and neutrophilia, mice treated with EHEC O157 OMVs also developed hyponatremia (**Figure 6C**), which has been identified as a predictor of HUS development in patients with EHEC infection (McKee et al., 2020) and a predictor of death in patients with HUS (Alconcher et al., 2018). Since mice treated with EHEC O157 OMVs exhibited hemoconcentration, neutrophilia, and hyponatremia, they might be utilized as a model for managing these complications in patients with EHEC-HUS. However, distinct mechanisms that likely underlie the pathophysiology of these disorders in mice and HUS patients need to be considered.

In conclusion, our data demonstrate that EHEC O157 OMVs meet the criteria for acting as EHEC virulence tools *in vivo* and for being involved in the pathogenesis of EHEC-HUS. We hypothesize that during human EHEC infection, both Stx2-containing OMVs and free Stx2 translocate from the intestine to the bloodstream and enter the kidneys, where they injure glomerular endothelial cells and tubular epithelial cells, which leads to acute kidney failure. The role of EHEC O157 OMVs in the pathogenesis of EHEC-HUS is further supported by their high production in the human intestine (Bauwens et al., 2017) and by their detection in the sera of patients with EHEC-HUS. The mode of transport of Stx2-carrying OMVs in the blood, and their interactions in EHEC-HUS pathogenesis with microvesicles and exosomes that transport free Stx2 (Ståhl et al., 2015; Karpman and Tontanahal, 2021), needs to be determined in future studies.

## Data Availability

The original contributions presented in the study are included in the article/**Supplementary Material**. Further inquiries can be directed to the corresponding author.
