# Supplementary material for: Enterohemorrhagic Escherichia coli O157 outer membrane vesicles administered by oral gavage cause renal tubular injury and acute kidney failure in mice
Source: Front Cell Infect Microbiol. 2025 Nov 24;15:1704731. doi: 10.3389/fcimb.2025.1704731 (PMC12682904; doi:10.3389/fcimb.2025.1704731)
Supplement: Supplementary file 1 [file DataSheet1.pdf]

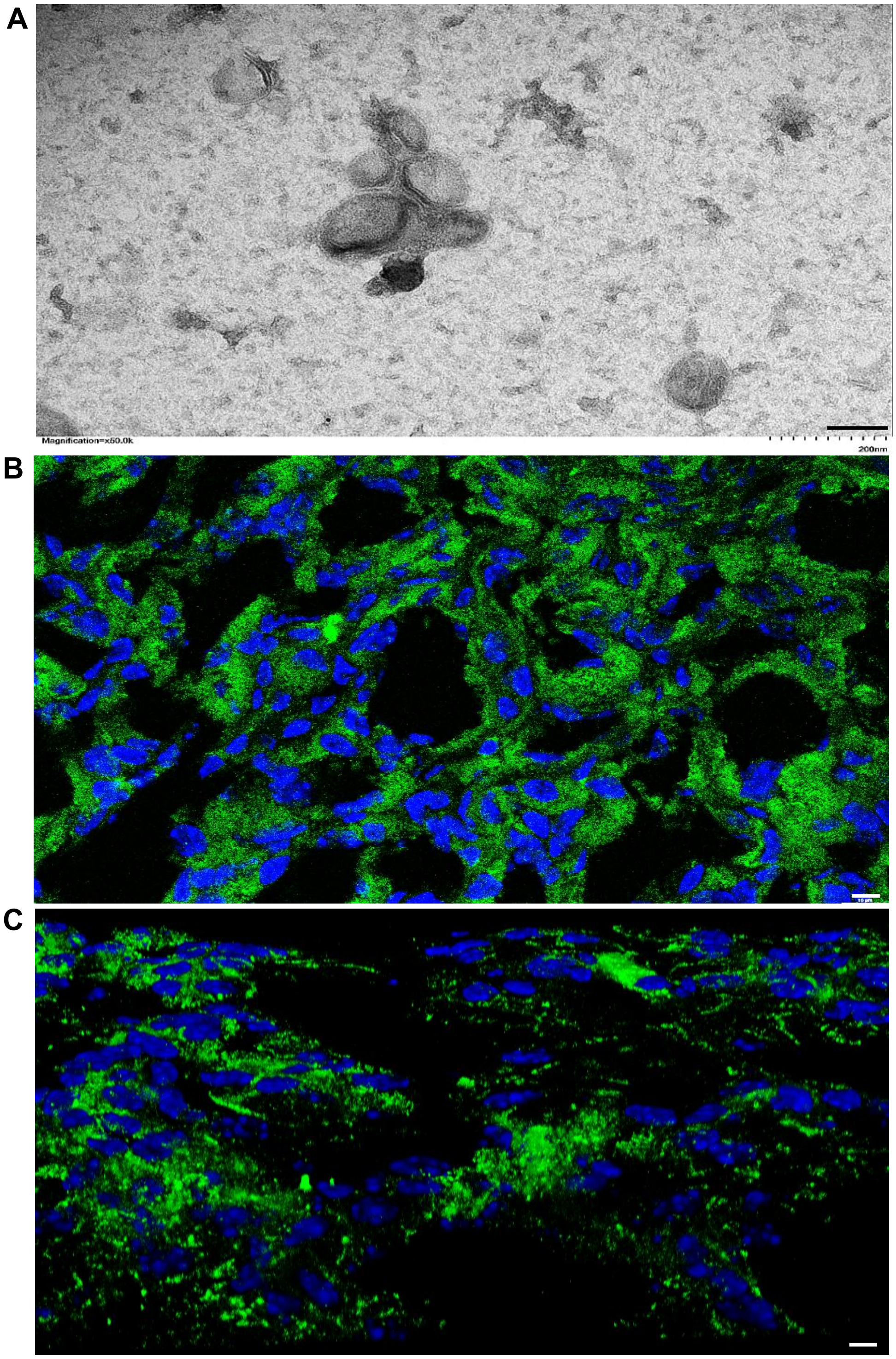

Supplementary Figure S1. (A) Specificity of immunogold staining in the mouse sera. Immunoelectron microscopy of serum sample from EHEC O157 OMV-treated mouse stained with gold-conjugated anti-rabbit IgG without anti-*E. coli* O157 LPS antibody. Scale bar is 100 nm. **(B, C)** Specificity of OMV immunofluorescence staining in the mouse kidneys. Cryosection from the kidney of EHEC O157 OMV-treated mouse stained with Cy3-conjugated goat anti-rabbit IgG without anti-*E. coli* O157 LPS antibody (OMVs), anti-CD324 rat monoclonal antibody and Alexa Fluor 488-conjugated goat anti-rat IgG (tubular epithelial cells), and DAPI (nuclei). Shown are **(B)** Z-stack, and **(C)** 3D image acquired using Leica LAS X 3D viewer. Scale bars are 10 µm.
