# Supplementary material for: Enterohemorrhagic Escherichia coli O157 outer membrane vesicles administered by oral gavage cause renal tubular injury and acute kidney failure in mice
Source: Front Cell Infect Microbiol. 2025 Nov 24;15:1704731. doi: 10.3389/fcimb.2025.1704731 (PMC12682904; doi:10.3389/fcimb.2025.1704731)
Supplement: Supplementary file 2 [file DataSheet2.pdf]

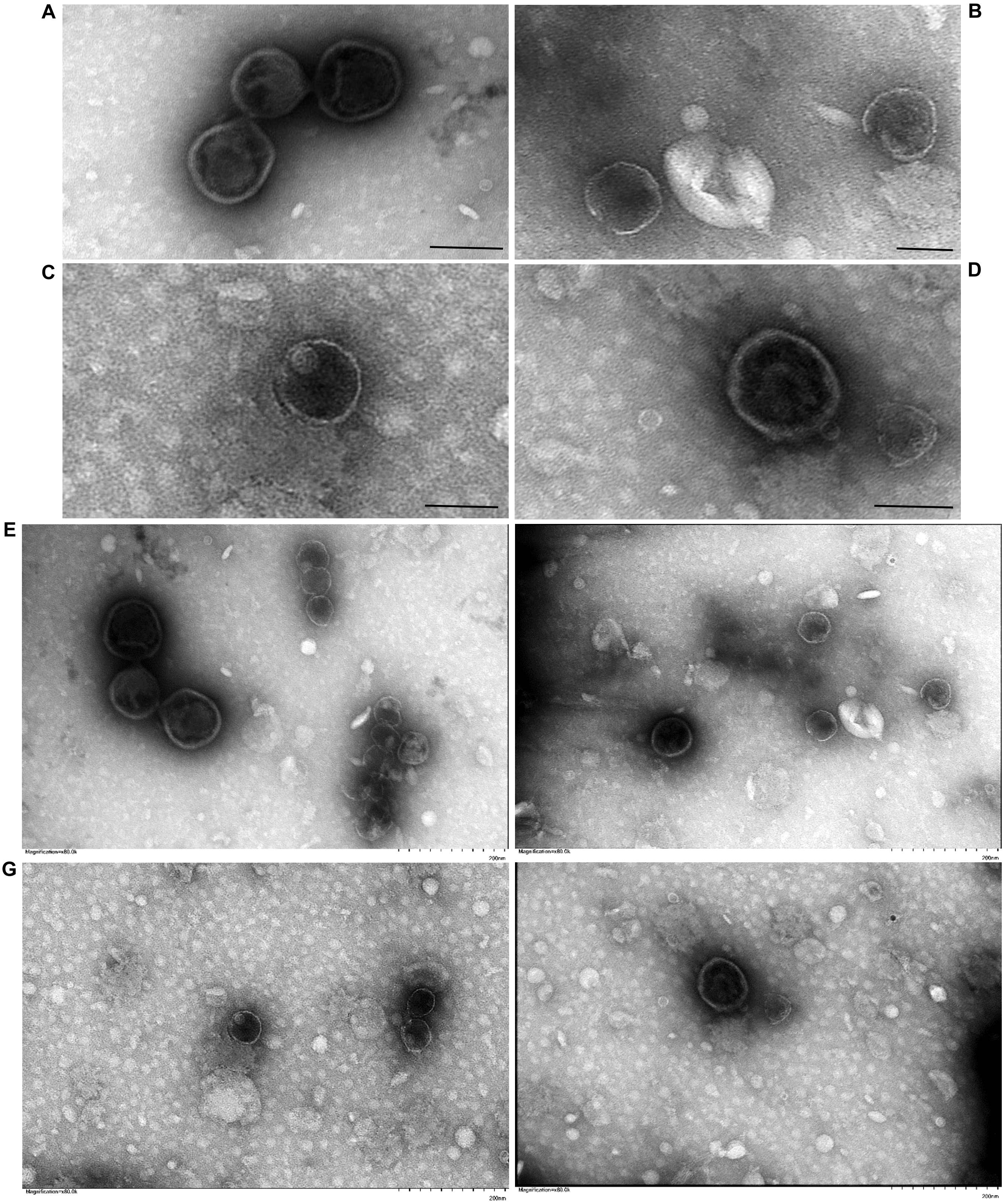

Supplementary Figure S2. Specificity of OMV immunogold staining in the sera of patients with EHEC-HUS. Image crops **(A-D)** and entire original images **(E-H)** of serum samples from two patients with EHEC O157-HUS stained with gold-conjugated goat anti-rabbit IgG without primary anti-*E. coli* O157 LPS antibody. **(A, B, E, F)** Patient 1. **(C, D, G, H)** Patient 2. Scale bars in panels **A-D** are 100 nm.
