# Supplementary material for: Enterohemorrhagic Escherichia coli O157 outer membrane vesicles administered by oral gavage cause renal tubular injury and acute kidney failure in mice
Source: Front Cell Infect Microbiol. 2025 Nov 24;15:1704731. doi: 10.3389/fcimb.2025.1704731 (PMC12682904; doi:10.3389/fcimb.2025.1704731)
Supplement: Supplementary file 3 [file DataSheet3.pdf]

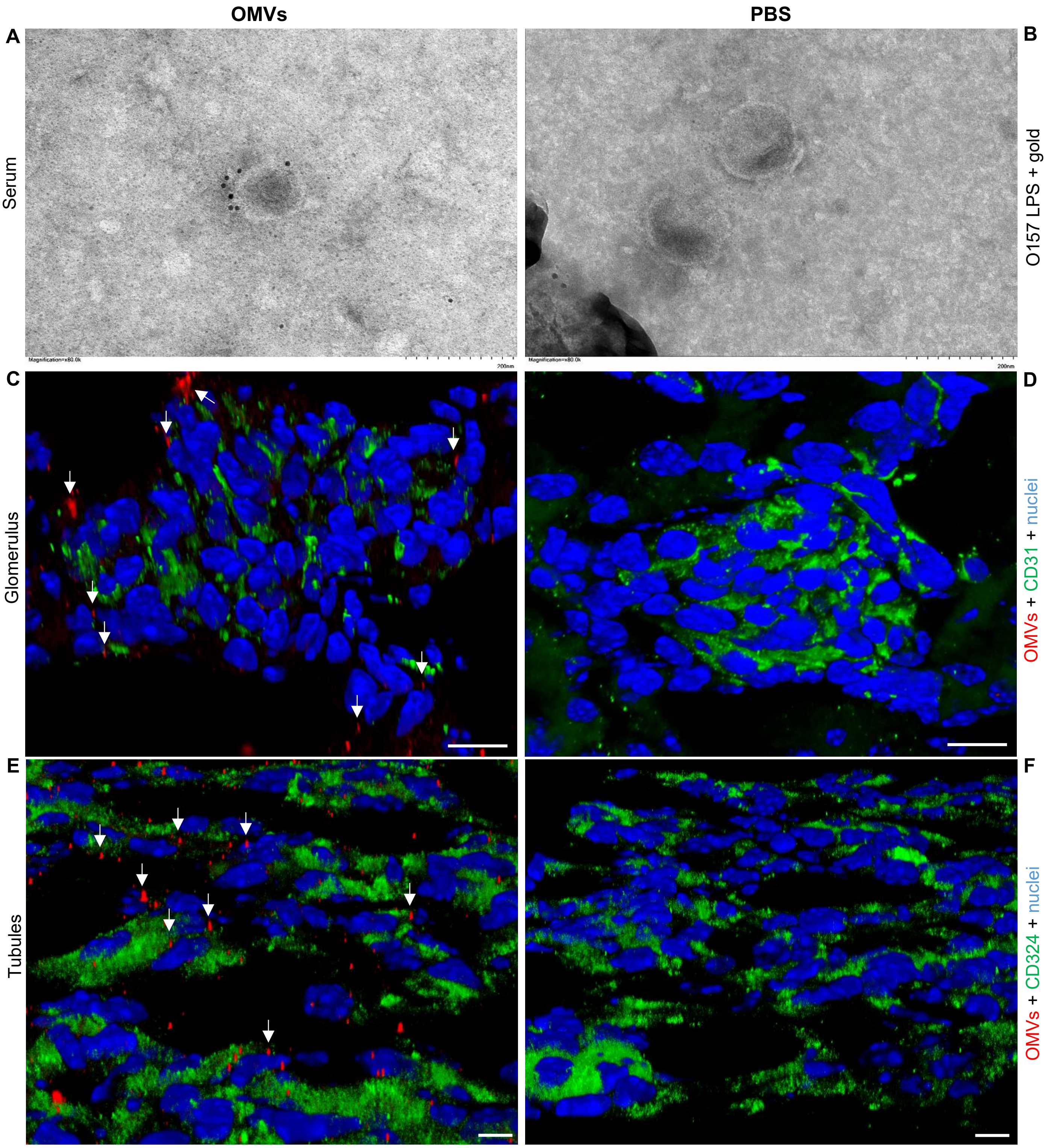

Supplementary Figure S3. Detection of EHEC O157 OMVs in the serum and kidneys of OMV-treated **(A, C, E)** and PBS-treated **(B, D, F)** mice. **(A, B)** Entire original images of immunoelectron microscopy of EHEC O157 OMVs in the mouse sera. Panels **(A, B)** correspond to panels **(A, B)** in Figure 1. **(C-F)** CLSM (3D images) of EHEC O157 OMVs in the glomeruli and tubules of OMV-treated **(C, E)** and PBS-treated **(D, F)** mice. EHEC O157 OMVs (red; depicted by arrows) were stained with rabbit anti-*E. coli* O157 LPS antibody and Cy3-conjugated goat anti-rabbit IgG, glomerular endothelial or tubular epithelial cells (green) with anti-CD31 or anti-CD324 rat antibody and Alexa Fluor 488-conjugated goat anti-rat IgG, and nuclei (blue) with DAPI. Scale bars 10 µm. Crops of CLSM images are shown. Entire original images and separate red, green, and blue channels are shown in Supplementary Figures S5A-D.
