# Supplementary material for: Enterohemorrhagic Escherichia coli O157 outer membrane vesicles administered by oral gavage cause renal tubular injury and acute kidney failure in mice
Source: Front Cell Infect Microbiol. 2025 Nov 24;15:1704731. doi: 10.3389/fcimb.2025.1704731 (PMC12682904; doi:10.3389/fcimb.2025.1704731)
Supplement: Supplementary file 5 [file DataSheet5.pdf]

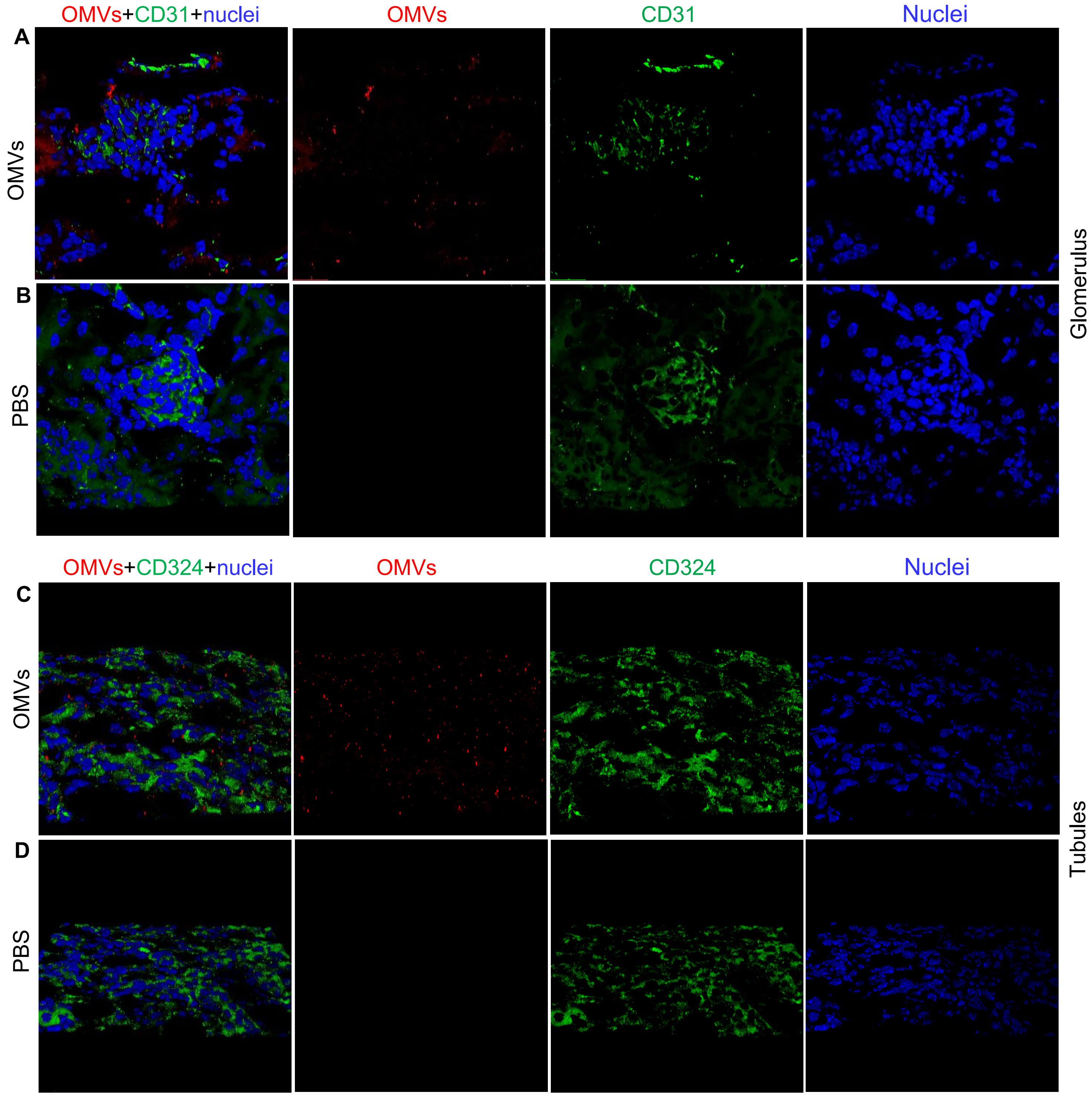

Supplementary Figure S5. Entire CLSM images (3D views) shown in Supplementary Figure S3 and separate red (OMV), green (CD31 or CD324), and blue (nuclei) channels. Rows A-D correspond to panels C-F in Supplementary Figure S3.
