# Supplementary material for: Enterohemorrhagic Escherichia coli O157 outer membrane vesicles administered by oral gavage cause renal tubular injury and acute kidney failure in mice
Source: Front Cell Infect Microbiol. 2025 Nov 24;15:1704731. doi: 10.3389/fcimb.2025.1704731 (PMC12682904; doi:10.3389/fcimb.2025.1704731)
Supplement: Supplementary file 6 [file DataSheet6.pdf]

A

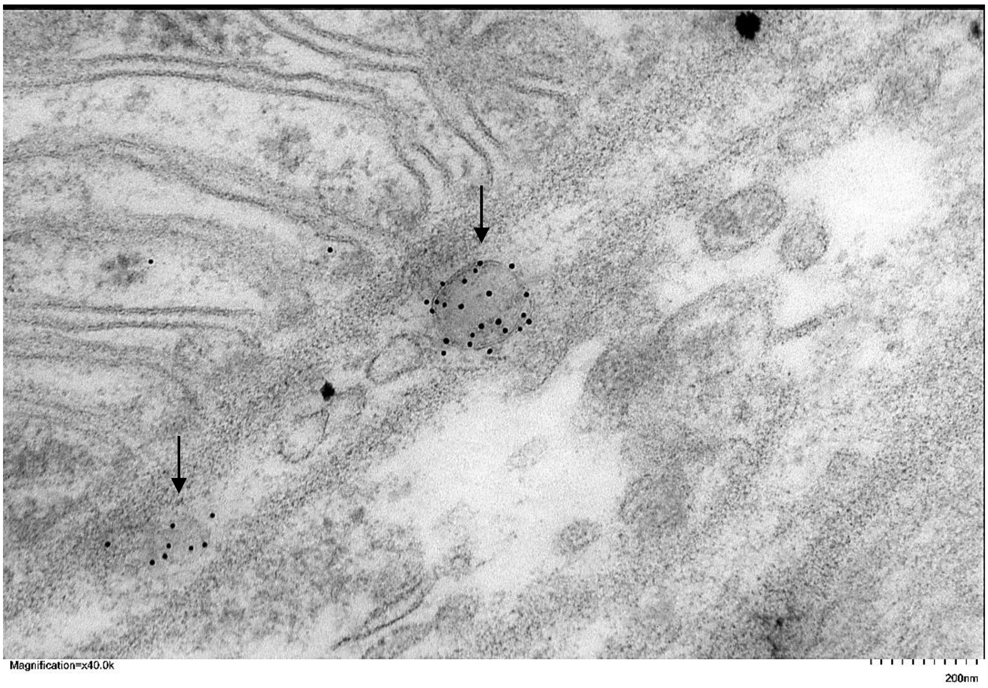

B

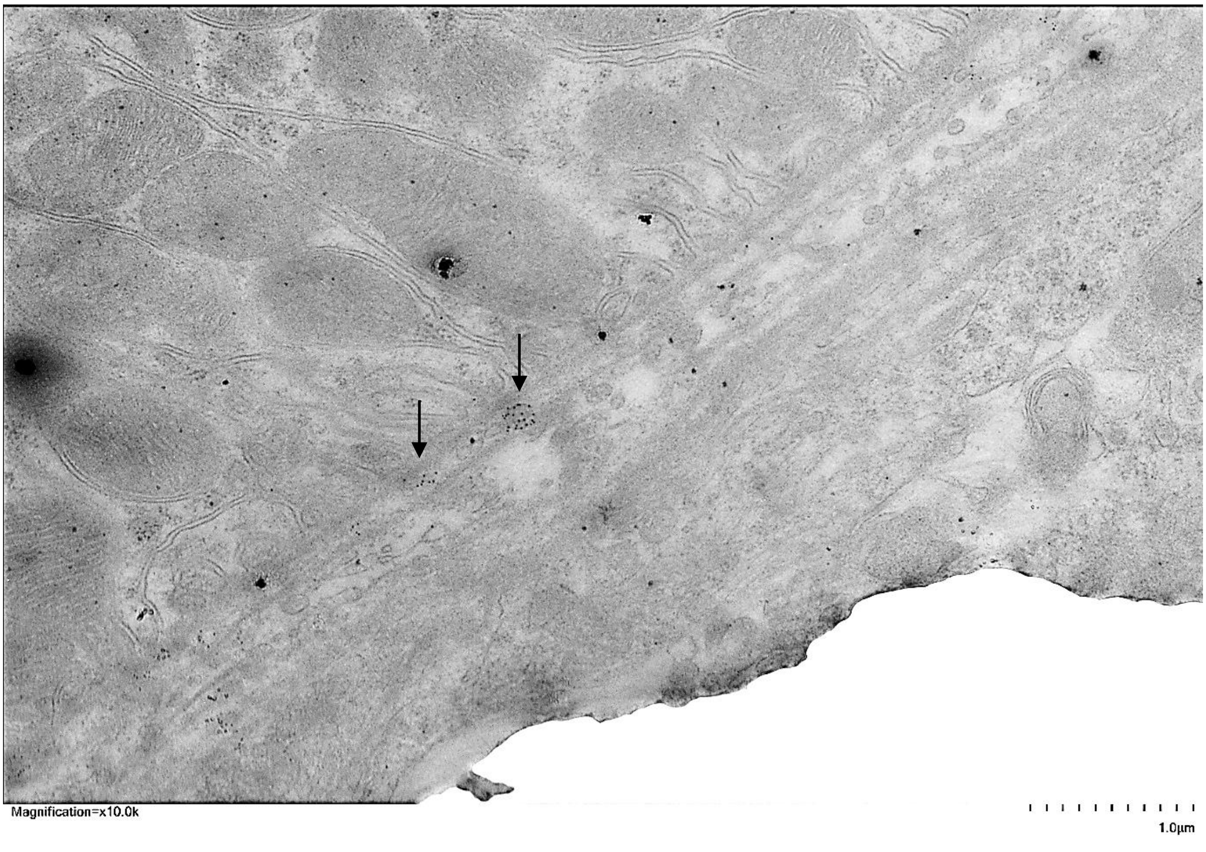

Supplementary Figure S6. Entire original images of EHEC O157 OMVs in the mouse kidneys detected by immunoelectron microscopy using rabbit anti-*E. coli* O157 LPS antibody and goat anti-rabbit IgG conjugated with colloidal gold 10 nm. Designations of entire images **(A)** and **(B)** correspond to the designations of crops **(A)** and **(B)** in Figure 2. OMVs are depicted by arrows.
