# Supplementary material for: Enterohemorrhagic Escherichia coli O157 outer membrane vesicles administered by oral gavage cause renal tubular injury and acute kidney failure in mice
Source: Front Cell Infect Microbiol. 2025 Nov 24;15:1704731. doi: 10.3389/fcimb.2025.1704731 (PMC12682904; doi:10.3389/fcimb.2025.1704731)
Supplement: Supplementary file 8 [file DataSheet8.pdf]

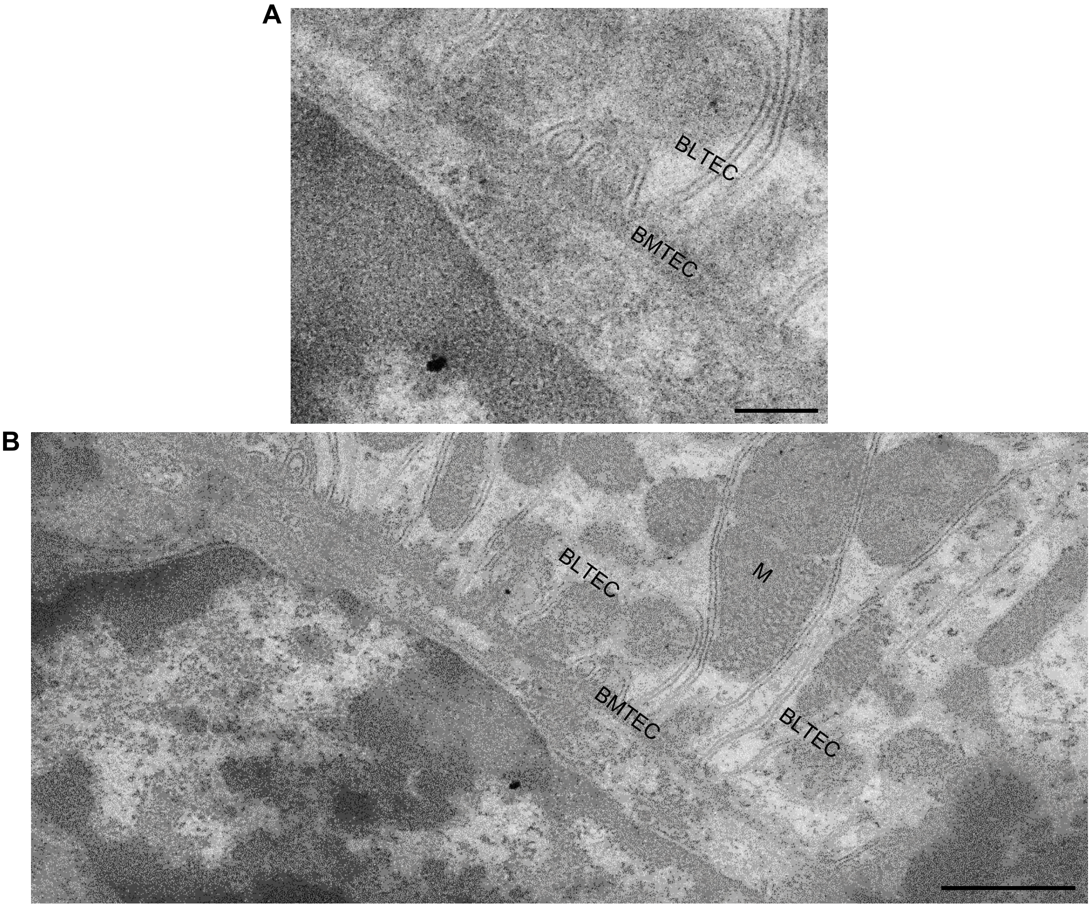

Supplementary Figure S8. EHEC O157 OMVs are absent from the kidneys of PBS-treated mice. Immunoelectron microscopy of ultrathin section from the kidney of a PBS-treated mouse stained with rabbit anti-*E. coli* O157 LPS antibody and goat anti-rabbit IgG conjugated with colloidal gold 10 nm. Immunogold-labeled structures corresponding to EHEC O157 OMVs were not detected in the kidney of a PBS-treated mice either at magnification 40.000x **(A)**, which allowed to visualize OMVs in the kidneys of OMV-treated mice (Figures 2A, C), or at magnification 10.000x **(B)**. Scale bars are 200 nm **(A)** or 1 µm **(B)**. BMTEC, basement membrane of tubular epithelial cell; BLTEC, basal labyrinth of tubular epithelial cell; M, mitochondria.
