# Supplementary material for: Enterohemorrhagic Escherichia coli O157 outer membrane vesicles administered by oral gavage cause renal tubular injury and acute kidney failure in mice
Source: Front Cell Infect Microbiol. 2025 Nov 24;15:1704731. doi: 10.3389/fcimb.2025.1704731 (PMC12682904; doi:10.3389/fcimb.2025.1704731)
Supplement: Supplementary file 9 [file DataSheet9.pdf]

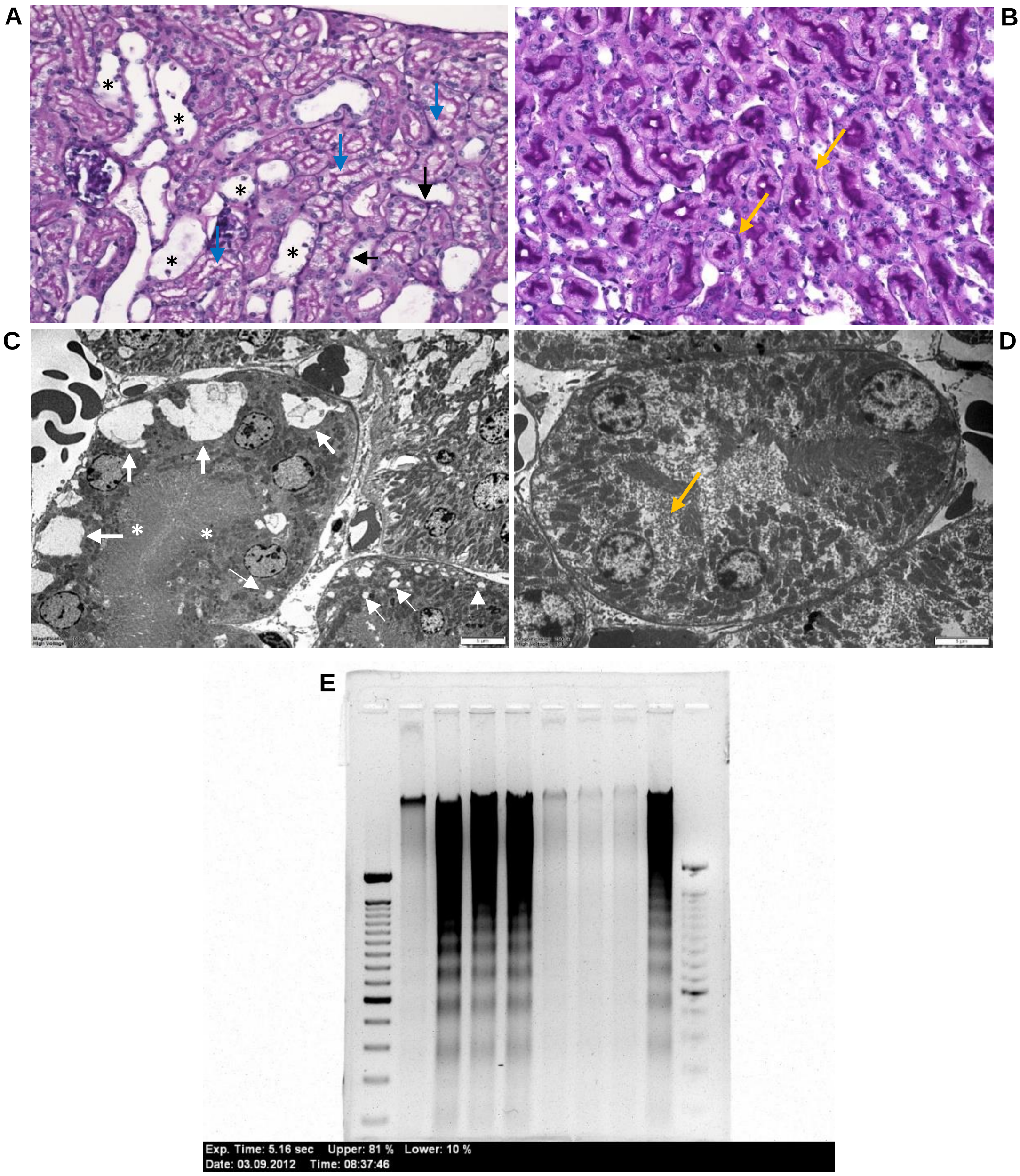

Supplementary Figure S9. EHEC O157 OMVs administered by oral gavage cause tubular epithelial damage and apoptosis. Histopathology **(A, B)** and electron microscopy **(C, D)** of the kidneys from mice administered 100 µg – 400 µg of OMVs **(A, C)** or PBS **(B, D)**. **(A)** Vacuolization of tubular epithelium (blue arrows), epithelial cell flattening (black arrows), and intraluminal detached cells (asterisks). **(B)** Normal tubules in a PBS-treated mouse (yellow arrows). Staining with PAS/alcian blue. Magnification 400x**.** **(C)** Macrovacuolization (thick white arrows) and microvacuolization (thin white arrows) of tubular epithelial cells with intact brush border (white asterisk). **(D)** Normal tubule in a PBS-treated mouse; yellow arrow indicates tubular epithelial cell. Scale bars 5 µm. **(E)** Entire original gel shown in Figure 4H (apoptosis in the kidneys of mice treated with EHEC O157 OMVs or PBS).
