# Supplementary material for: Enterohemorrhagic Escherichia coli O157 outer membrane vesicles administered by oral gavage cause renal tubular injury and acute kidney failure in mice
Source: Front Cell Infect Microbiol. 2025 Nov 24;15:1704731. doi: 10.3389/fcimb.2025.1704731 (PMC12682904; doi:10.3389/fcimb.2025.1704731)
Supplement: Supplementary file 10 [file DataSheet10.pdf]

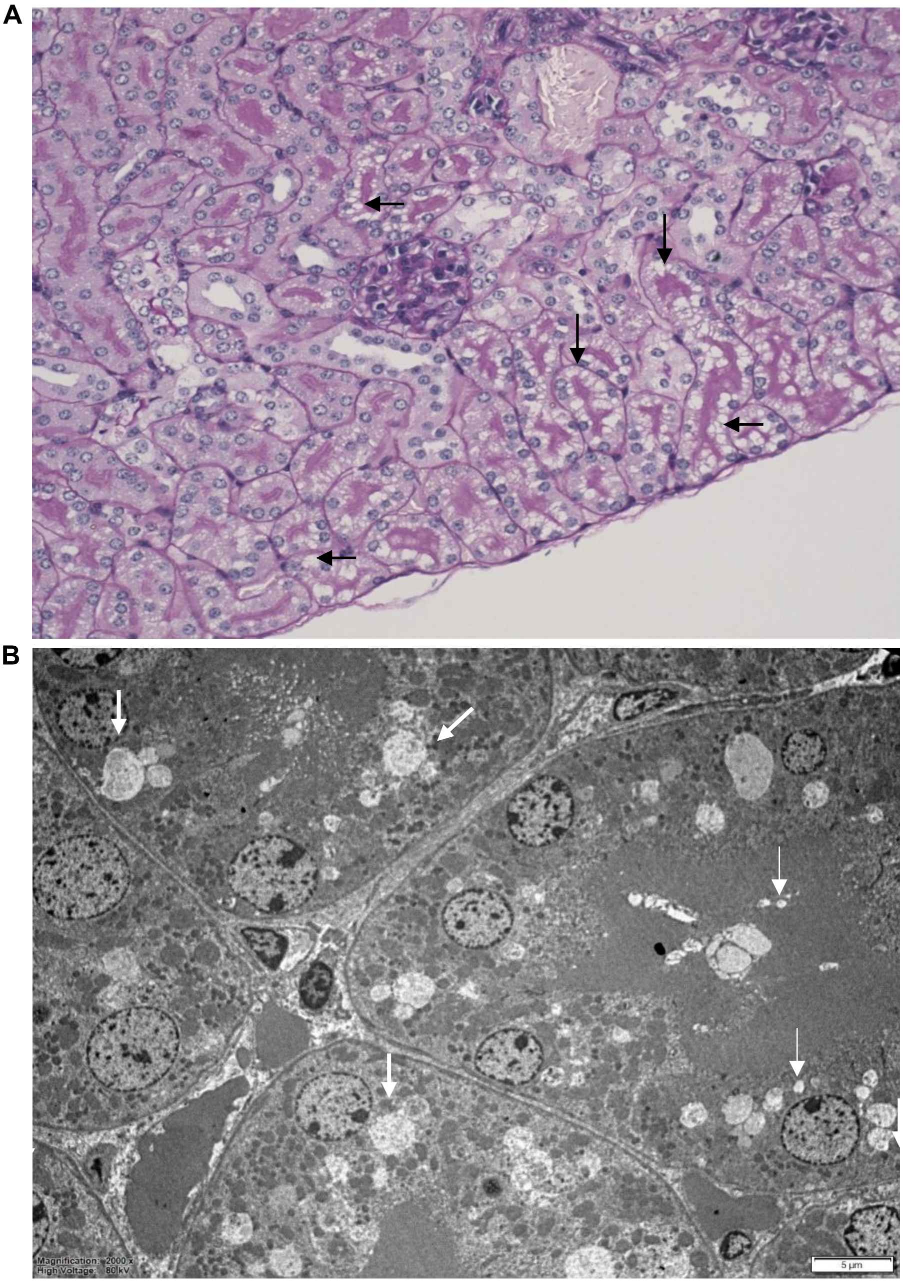

Supplementary Figure S10. Vacuolization of the kidney tubular epithelium in a mouse administered 25 µg of EHEC O157 OMVs. **(A)** Paraffin section stained with PAS/alcian blue; vacuoles are indicated by black arrows; magnification 400x**.** **(B)** Transmission electron microscopy showing the presence of microvacuoles (thin white arrows) and macrovacuoles (thick white arrows) in tubular epithelial cells; scale bar 1 µm.
