# Supplementary material for: Enterohemorrhagic Escherichia coli O157 outer membrane vesicles administered by oral gavage cause renal tubular injury and acute kidney failure in mice
Source: Front Cell Infect Microbiol. 2025 Nov 24;15:1704731. doi: 10.3389/fcimb.2025.1704731 (PMC12682904; doi:10.3389/fcimb.2025.1704731)
Supplement: Supplementary file 11 [file DataSheet11.pdf]

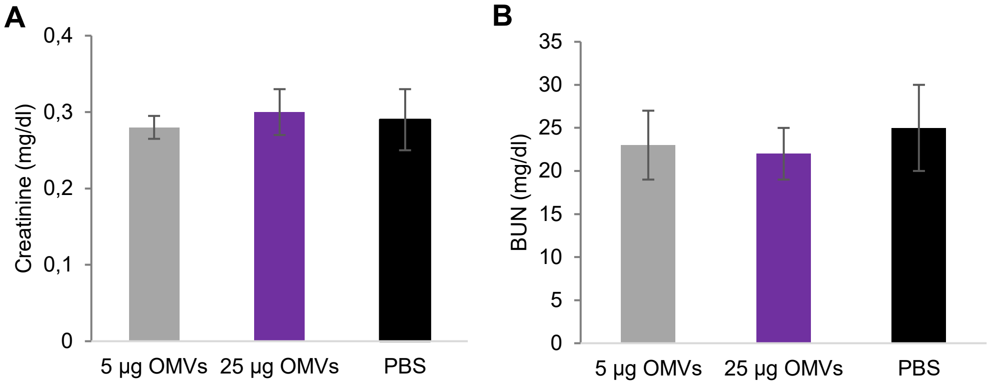

Supplementary Figure S11. Serum concentrations of **(A)** creatinine and **(B)** blood urea nitrogene (BUN) in mice that received 5 µg or 25 µg of EHEC O157 OMVs and did not show any tubular epithelial damage in histopathological and electron microscopic examinations. Data are means ± standard deviations from the measurements in OMV-treated mice and PBS-treated mice, respectively.
