# Supplementary material for: Enterohemorrhagic Escherichia coli O157 outer membrane vesicles administered by oral gavage cause renal tubular injury and acute kidney failure in mice
Source: Front Cell Infect Microbiol. 2025 Nov 24;15:1704731. doi: 10.3389/fcimb.2025.1704731 (PMC12682904; doi:10.3389/fcimb.2025.1704731)
Supplement: Supplementary file 12 [file DataSheet12.pdf]

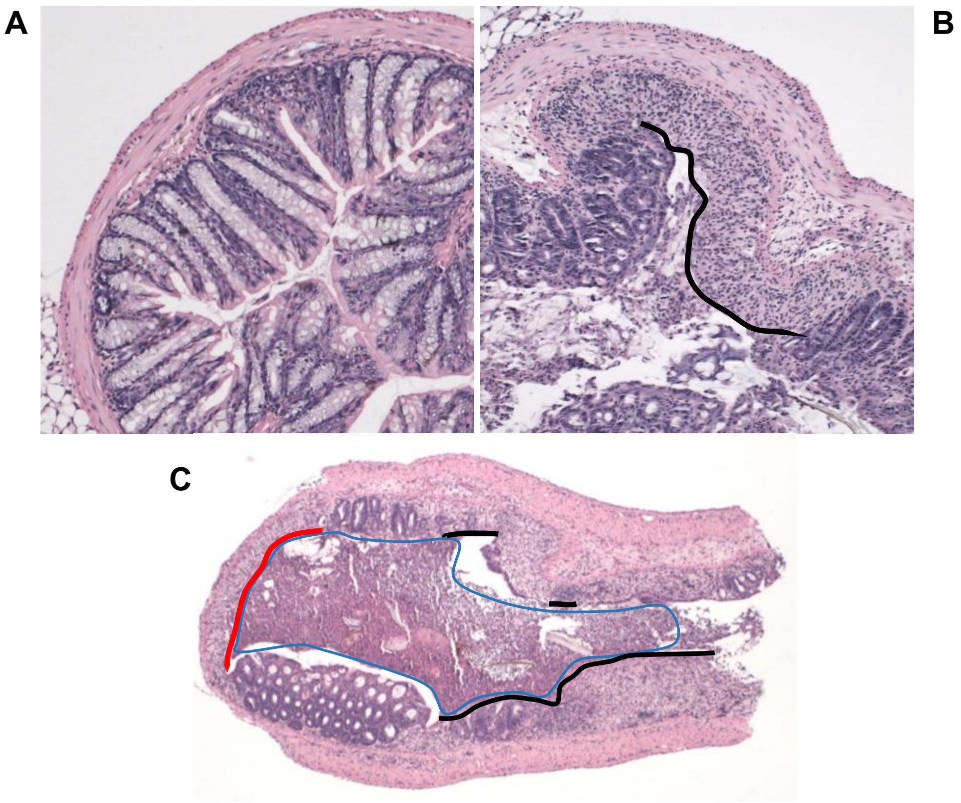

Supplementary Figure S12. EHEC O157 OMVs administered by oral gavage induce colitis in mice. Histopathology of paraffin sections of the colon from mice administered PBS **(A)** or EHEC O157 OMVs **(B, C)**. Sections were stained with hematoxylin eosin. **(B)** Erosion in the colon from a mouse administered 100 µg of EHEC O157 OMVs (area marked with a black line). **(C)** Erosions (areas marked with a black line), ulceration (area marked with a red line), and inflammatory infiltration (area marked with a blue line) in the colon from a mouse administered 200 µg of EHEC O157 OMVs. Magnification 400x **(A, B)** or 200x **(C)**.
