# Supplementary material for: Enterohemorrhagic Escherichia coli O157 outer membrane vesicles administered by oral gavage cause renal tubular injury and acute kidney failure in mice
Source: Front Cell Infect Microbiol. 2025 Nov 24;15:1704731. doi: 10.3389/fcimb.2025.1704731 (PMC12682904; doi:10.3389/fcimb.2025.1704731)
Supplement: Supplementary file 13 [file DataSheet13.pdf]

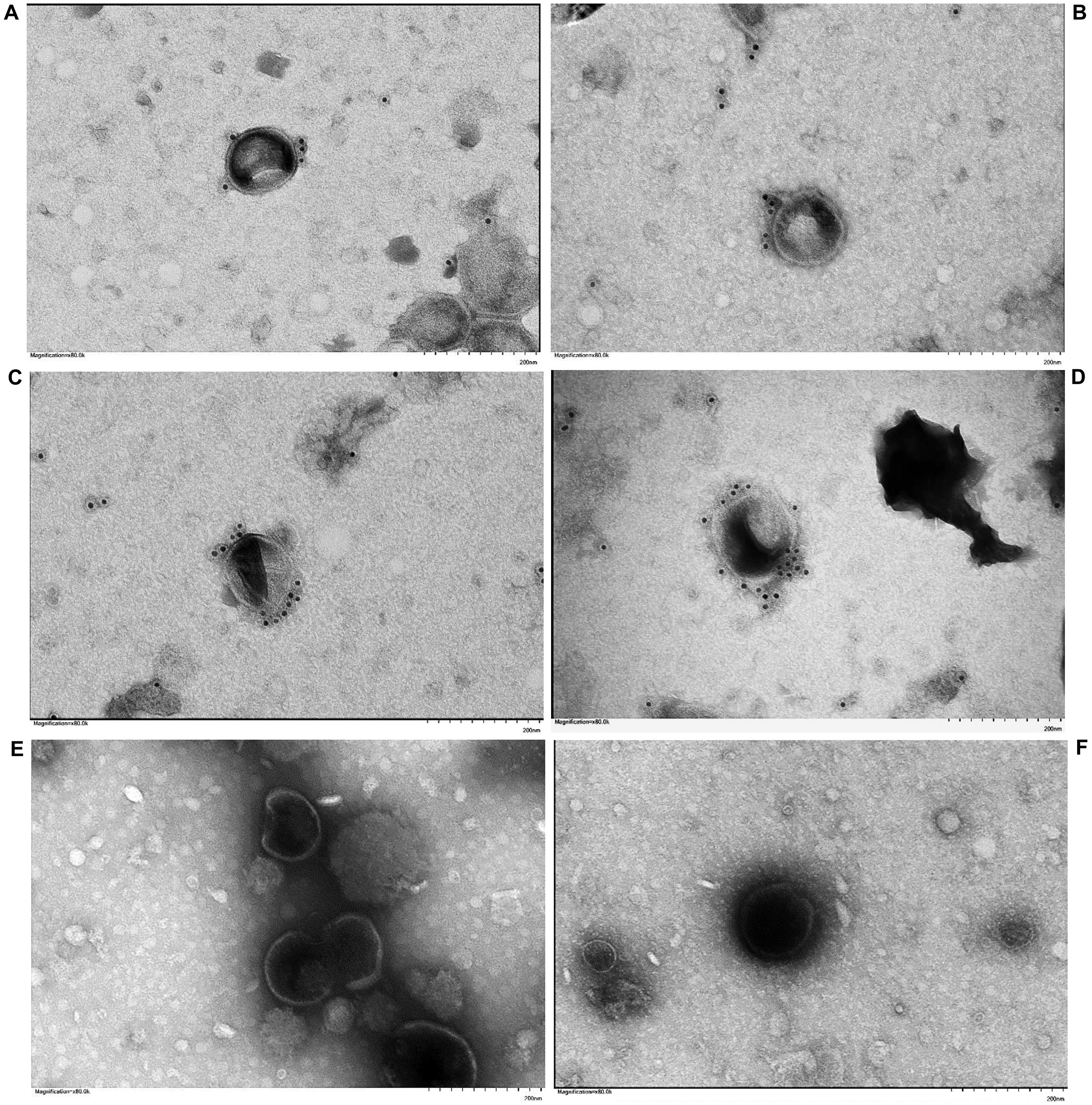

Supplementary Figure S13. Entire original images showing detection of EHEC O157 OMVs in the sera of patients with EHEC O157-associated HUS **(A-D)** and of a control EHEC O157-negative person **(E, F)** by immunoelectron microscopy with anti-*E. coli* O157 LPS antibody and goat anti-rabbit IgG conjugated with colloidal gold 10 nm. The designations of entire original images **(A-F)** correspond to the designations of crops **(A-F)** in Figure 8**.**
