# Supplementary material for: Enterohemorrhagic Escherichia coli O157 outer membrane vesicles administered by oral gavage cause renal tubular injury and acute kidney failure in mice
Source: Front Cell Infect Microbiol. 2025 Nov 24;15:1704731. doi: 10.3389/fcimb.2025.1704731 (PMC12682904; doi:10.3389/fcimb.2025.1704731)
Supplement: Supplementary file 14 [file DataSheet14.pdf]

Supplementary Table S1. Health scoring criteria for mice administered EHEC O157 OMVs or PBS (modified from Dennhardt et al., 2018)

Score	Disease severity	Activity	Reaction	Fur	Posture	Neurological symptoms
0	No signs of disease	strong	curious, fast movements	shiny, even	normal	none
1	Low-grade	lower activity	appropriate reaction	blunt, adjacent	normal	none
2	Mid-grade	markedly reduced, apathy	delayed reaction	blunt, slightly ruffled	slightly hunched	ataxia
3	High-grade	lethargic, no movements	none	blunt, strong piloerection	strong hunch	tremor
